# MR-labelled liposomes and focused ultrasound for spatiotemporally controlled drug release in triple negative breast cancers in mice

**DOI:** 10.7150/ntno.52168

**Published:** 2021-01-01

**Authors:** Maral Amrahli, Miguel Centelles, Paul Cressey, Martynas Prusevicius, Wladyslaw Gedroyc, Xiao Yun Xu, Po-Wah So, Michael Wright, Maya Thanou

**Affiliations:** 1 School of Cancer & Pharmaceutical Sciences, King's College London, U.K.; 2 Department of Academic Surgery, Imperial College London, U.K.; 3 Department of Chemical Engineering, Imperial College London, U.K.; 4 Department of Neuroimaging, King's College London, U.K.

**Keywords:** liposome, MRI, focused ultrasound, doxorubicin, triple-negative breast cancer

## Abstract

**Rationale:** Image-guided, triggerable, drug delivery systems allow for precisely placed and highly localised anti-cancer treatment. They contain labels for spatial mapping and tissue uptake tracking, providing key location and timing information for the application of an external stimulus to trigger drug release. High Intensity Focused Ultrasound (HIFU or FUS) is a non-invasive approach for treating small tissue volumes and is particularly effective at inducing drug release from thermosensitive nanocarriers. Here, we present a novel MR-imageable thermosensitive liposome (iTSL) for drug delivery to triple-negative breast cancers (TNBC).

**Methods:** A macrocyclic gadolinium-based Magnetic Resonance Imaging (MRI) contrast agent was covalently linked to a lipid. This was incorporated at 30 mol% into the lipid bilayer of a thermosensitive liposome that was also encapsulating doxorubicin. The resulting iTSL-DOX formulation was assessed for physical and chemical properties, storage stability, leakage of gadolinium or doxorubicin, and thermal- or FUS-induced drug release. Its effect on MRI relaxation time was tested in phantoms. Mice with tumours were used for studies to assess both tumour distribution and contrast enhancement over time. A lipid-conjugated near-infrared fluorescence (NIRF) probe was also included in the liposome to facilitate the real time monitoring of iTSL distribution and drug release in tumours by NIRF bioimaging. TNBC (MDA-MB-231) tumour-bearing mice were then used to demonstrate the efficacy at retarding tumour growth and increasing survival.

**Results**: iTSL-DOX provided rapid FUS-induced drug release that was dependent on the acoustic power applied. It was otherwise found to be stable, with minimum leakage of drug and gadolinium into buffers or under challenging conditions. In contrast to the usually suggested longer FUS treatment we identified that brief (~3 min) FUS significantly enhanced iTSL-DOX uptake to a targeted tumour and triggered near-total release of encapsulated doxorubicin, causing significant growth inhibition in the TNBC mouse model. A distinct reduction in the tumours' average T_1_ relaxation times was attributed to the iTSL accumulation.

**Conclusions:** We demonstrate that tracking iTSL in tumours using MRI assists the application of FUS for precise drug release and therapy.

## Introduction

More than 40 years ago it was suggested that the effectiveness of anticancer drugs might be enhanced by the application of localised mild hyperthermia 1. Recent technological advances allow for the application of heat to deep seated tissues, with high spatial precision and accurate thermal control. High Intensity Focused Ultrasound (HIFU or FUS) is the most accurate non-invasive hyperthermia method 2. Clinical FUS was developed to deposit ultrasound energy within small tissue volumes 3. Its extension to Magnetic Resonance guided (MRg) FUS allows for highly accurate spatial localisation and is clinically used for precise ablative treatment of solid tumours and neurological disorders 4. A less clinically investigated application is the use of MR-guided FUS hyperthermia for targeted drug delivery using thermoresponsive liposomes 5,6.

Liposomes are in widespread clinical use as nanocarriers that offer a high level of multifunctionality and versatility 7. Thermosensitive liposomes (TSL) have been developed as drug delivery systems able to release their cargo almost instantly when warmed to ~42 °C 8,9. TSL have been tested in small and large animals and have shown great efficiency when combined with hyperthermia 10,11. A potentially valuable (but not yet fully developed) modification would be the inclusion of MRI contrast agents (CA) to allow for real-time image guidance, uptake tracking, and monitoring of the drug release within a lesion 12-14. Such MR-labelled carriers also offer anatomical guidance, feedback, and spatiotemporal control of the applied FUS - offering the opportunity of real-time treatment for large tumours and potentially their metastases 15.

It has been shown that FUS can be combined with TSL loaded with gadolinium-based paramagnetic MRI contrast agents 16. A study by Kono *et al.* has reported on gadolinium DOTA (1,4,7,10-tetraazacyclododecane-1,4,7,10-tetraacetic acid) chelates introduced in a dendron structure attached to the lipid membrane of liposomes coated with thermossensitive polymer 12. But usually gadolinium-macrocyclic CA (FDA approved and common in clinical imaging) are encapsulated into the aqueous TSL core 17. The TSL release these small molecules after heating, giving an MR-visible confirmation of any concurrent drug release - a technique dubbed 'dose painting' 18. The effect is transient since the freed CA rapidly diffuses away from the tumour. Lipid membrane-bound CA have the advantage here as they can be monitored throughout a treatment and for longer timeframes 12.

ThermoDox® is a doxorubicin-bearing TSL and was the first 'responsive' nanocarrier that moved to clinical trials 9. A recently completed Phase-III (NCT00617981) evaluated its efficacy in combination with radiofrequency ablation in patients with hepatocellular carcinoma 19, while TARDOX is a published Phase-I that demonstrated the safety of using FUS to trigger drug release 20. This showed that 60 min of FUS treatment significantly increased doxorubicin concentration in tumours. We previously presented dual-labelled (near-infrared fluorescence and MRI) thermosensitive liposomes for imaging (iTSL) as a tool that provides insight on the mechanism of drug release and the temporal and spatial distribution of drug nanocarriers in tumours 21,22. Near-infrared fluorescence (NIRF) imaging tracked the liposomes in real time, allowing assessment of their distribution and this information was used to decide the time and duration of FUS treatments. We demonstrated that FUS caused an increase in iTSL uptake to tumours and triggered coordinated drug release (using topotecan). Surprisingly, only brief FUS treatments were needed to significantly modify liposome distribution and induce rapid drug release 21,22.

Here, we investigate the ability of iTSL loaded with doxorubicin (iTSL-DOX) to be tracked by MRI in mice and assess if brief FUS treatments can significantly improve the drug's anti-tumour efficacy. We modify the composition of a lysolipid-containing TSL with a gadolinium-DOTA lipid to allow image-based tracking in tumours post-injection. We synthesised and incorporated the MR-labelled lipid (Gd.DOTA.DSA) in a liposome bilayer at almost 30 mol% of the lipid composition and investigated the effects of this on thermo-sensitivity and the ability to encapsulate/release doxorubicin. After optimisation of the composition, we assessed iTSL-DOX for drug and Gd^3+^ leakage and for stability and thermal release performance after long term storage (3 months). Using iTSL-DOX in an acoustic gel block embedded capillary tube model, we demonstrate that drug release can be instantaneous and is strongly dependent on the applied FUS power. To allow for the tracking of iTSL-DOX *in vivo* a small amount of lipid-conjugated NIRF probe (CF750-DSDA; 0.05 mol%) was also included. This label provided valuable information during development, although NIRF is unlikely to be used in the clinic due to limited tissue penetration. NIRF-imaging in live mice showed iTSL-DOX accumulation and doxorubicin release in tumours during the application of FUS. Efficacy on tumours was demonstrated on a triple-negative breast cancer (TNBC; MDA-MB-231) murine model. The potential advantages of MRI tracking are shown by assessing liposome effects on phantom and tumour T_1_ relaxivity (a measure of contrast enhancement).

## Material and Methods

### Lipid synthesis

Gadolinium (III) 2-(4,7-bis-carboxymethyl-10-[(*N*, *N*-distearylamidomethyl-*N'*-amidomethyl]-1,4,7,10-tetraazacyclododec-1-yl) acetic acid (Gd.DOTA.DSA; MR-labelled lipid) and *N*`-CF750-*N*,*N*-distearylamidomethylamine (CF750.DSA; NIRF-labelled lipid) were synthesised, purified, and confirmed according to our previous report 21 (Scheme S1; CF750 succinimidyl ester (Biotium, CA, USA) was previously called XL750-NHS).

### Preparation of iTSL-DOX

1,2-Dipalmitoyl-*sn*-glycero-3-phosphocholine (DPPC; 16:0 PC), 1,2-distearoyl-*sn*-glycero-3-phosphocholine (DSPC; 18:0 PC), 1-stearoyl-*sn*-glycero-3-phosphocholine (MSPC; 18:0 lyso-PC) and (*ω*-methoxy-polyethyleneglycol^2000^)-*N*-carboxy-1,2-distearoyl-*sn*-glycero-3-phosphoethanolamine (DSPE-PEG^2000^) were purchased from Avanti Polar Lipids (AL, USA) or Sigma Aldrich (MO, USA). Lipids were stored at -20 °C as aliquots of 10-20 mg/mL in chloroform, methanol or methanol:chloroform 1:1 (v/v) according to their solubility. Appropriate amounts of each were combined to give a mixture of Gd.DOTA.DSA/DPPC/DSPC/MSPC/DSPE-PEG^2000^/CF750.DSA at 30/54/5/5/6/0.05 mol% and 30 mg total lipid per batch. The solvent was removed using a rotary evaporator and the resulting film dried overnight *in vacuo*. Further preparation stages were carried out in a sterilised fume hood with all equipment and materials being single use, sterilised by autoclave, or wiped down with ethanol. Buffers were sterilised by filtration (0.2 µm).

Each lipid film was hydrated with 1 mL of loading buffer (300 mM ammonium phosphate aq.; pH 4.0) and fragmented using repeated (~10×) freeze/thaw from liquid nitrogen to a water sonication bath at 53 °C, followed by further sonication, and extrusion 3× through a 100 nm pore polycarbonate membrane using a gas powered extruder (LIPEX, Northern Lipids, Canada) heated to 52 °C. The iTSL was then exchanged to storage buffer (50 mM HEPES aq. with 5 w% glucose; pH 7.4) using a PD10 size-exclusion column (Amersham, UK). They were loaded with doxorubicin using a thermocycler to provide accurate temperatures. Concentrated doxorubicin HCl aq. (stored frozen) was added to 1.2 mg/mL, then incubated at 38.7 °C for 1 h 50 m. Sterility was checked by inoculating LB-agar plates with 100-fold diluted iTSL-DOX and incubation overnight at 37 °C. No bacterial colonies were seen, unlike plates inoculated with non-sterile storage buffer.

### Dynamic Light Scattering (DLS)

iTSL-DOX batches were routinely assessed using a Nanoseries Nano ZS (Malvern Panalytical, UK). Samples were diluted 1:20 (v/v) using storage buffer at 25 °C and size modelling used default solute and particle parameters.

### Doxorubicin quantification by HPLC

iTSL-DOX absorbance and fluorescence profiles were collected using an Infinite 200 Pro plate reader (Tecan, Switzerland). Expected absorbance peaks were seen at 480 nm (doxorubicin), 750 nm (CF750 NIRF dye) and fluorescence peaks at Ex_480_/Em_590_ (doxorubicin), Ex_750_/Em_800_ (CF750). Dilute iTSL-DOX shows a 3-5× increase in doxorubicin fluorescence intensity on incubation at temperatures above 40 °C, due to release of encapsulated drug and resulting de-quenching. The NIR fluorescence of the iTSL is not affected by this.

Total doxorubicin concentration was assessed by reverse-phase HPLC using an Agilent 1100 equipped with a multi-wavelength diode array detector, a 1260 Infinity fluorescence detector (all from Agilent, CA, USA), and a 5 cm Hypersil C18 5 µm reverse-phase column (Thermo Fisher, MA, USA). Solvents were: water with 0.1 v% trifluoroacetic acid (A) and acetonitrile (B). Gradient was: 0 min 0% B, 1.5 min 0%, 5 min 50%, 6 min 50%, 7 min 0%, 8.5 min 0% and a flow rate of 3.5 mL/min. Doxorubicin gave a single fluorescence peak (4.1 min) the area being used to estimate concentration after calibration (0-200 ng range; R 0.999) against a doxorubicin-HCl European Pharmacopoeia Reference Standard.

During loading optimisation, the lipid:drug ratio was assessed by gel-filtration HPLC using a Tricorn 5/100 column (GE Life Sciences, PA, USA) packed with Sephadex G20 slurry and run in water at 1 mL/min. Total lipid concentration determination used a modified Stewart assay: iTSL-DOX samples (50 µL) were mixed with water (150 µL) and MeOH:CHCl_3_, 1:1 (v/v; 200 µL) then vortex mixed giving an emulsion. This was centrifuged (4000 g; 2 min) to fully separate the organic and aqueous layers. An aliquot (70 µL) of the organic layer was combined with Stewart reagent (5 µL; FeCl_3_/NH_4_SCN aq.), emulsified and centrifuged again. Finally, an organic aliquot (50 µL) was transferred to a glass 96-well plate and the 455 nm absorbance was measured. Calibration was against matched lipid suspensions prepared with sonication alone to avoid extrusion loses.

### Gadolinium quantification by total-reflection X-ray fluorescence (TXRF)

This was performed on a PICOFOX™-S2 (Bruker, MA, USA) 23 with samples mixed 1:1 (v/v) with diluted gallium aq. (TraceCERT^®^ certified reference) as an internal standard and using Ultrapure water and trace metal analysis grade reagents. Tissues were digested with nitric acid (150 µL; 68 w% aq.) and hydrogen peroxide (50 µL; 30 w% aq.) in tightly sealed plastic tubes at 70 °C overnight, while heparin-treated blood and liposome samples were analysed directly. Sample/standard mixtures were homogenised, 5-10 µL applied onto siliconised quartz disc sample carriers (Bruker Nano GMbH, Germany) and oven dried at 70 °C. TXRF results were collected in triplicate over 1000 s using excitation settings of 50 kV and 600 mA. Spectra were inspected and all significant peaks identified, prior to deconvolution using PICOFOX™ V 7.5.3.0 (Bruker Nano GmbH).

### iTSL-DOX thermosensitivity

Assessment of lipid membrane thermosensitivity used liquid phase differential scanning calorimetry (DSC, TA Instruments, DE, USA) to record at least two rounds of heating/cooling cycles (25-70 °C at 1 °C/min; 3 atm) from samples of each iTSL-DOX batch against degassed plain buffer.

Thermally-induced doxorubicin release was assessed by the increase in Ex_480_/Em_590_ fluorescence seen as it escapes from the highly self-quenched encapsulated environment after incubation >42 °C. Studies used separate samples (100 µL; triplicate) diluted 1:100 (v/v) in storage buffer, or 50 v% fetal bovine serum (FBS) as a blood analogue. Fluorescence intensity readings were normalised against iTSL-DOX, unheated and also after 5 min at 50 °C to assess release%.

### iTSL-DOX gadolinium retention

The potential for 'leakage' of Gd^3+^ from chelation to Gd.DOTA.DSA was investigated using a dialysis assay. This measured the escape of Gd^3+^ from an inner chamber of a Slide-A-Lyzer (MINI Dialysis Device, 10K MWCO, Thermo Scientific) containing either iTSL or 0.2 mg/mL aq. gadolinium standard (TraceCERT®) to an outer cuvette containing either Ultrapure water or 50 v% FBS water (at 4 °C to avoid protein aggregation). The cuvettes were equipped with small magnetic beads, placed on a magnetic stirrer then 10 μL samples taken over 48 h and analysed by TXRF as above. Doxorubicin was omitted as it blocked the dialysis membrane.

### FUS-induced doxorubicin release

A gel block embedded capillary model was developed that allowed for fluorescence visualisation of doxorubicin release from iTSL-DOX while under FUS using a preclinical small animal FUS system (Therapy and Imaging Probe System, 'TIPS'; Philips Research, Netherlands; Figure S1 -2).

Optically transparent polyacrylamide gel with suitable acoustic properties was cast into cylindrical blocks. A fine plastic flow-tube (0.8 mm inner diameter) was placed within the gel block positioned to pass through the focal volume of the transducer. A fine-wire thermocouple (T150A; Linton Instrumentation, U.K.) was threaded through the flow-tube such that the sensing tip was placed next to the focus. The other end of the flow-tube was connected to a syringe of iTSL-DOX (200-fold dilution into storage buffer) pumped by a driver at up to 100 µL/min. A pad of acoustic foam was placed below the gel block to absorb the post-focus FUS and prevent the formation of standing waves. The gel block was then immersed in a water bath at ~35 °C and illuminated from the sides, either by white light or 3× blue LED (460 nm peak, 10 W; LED Engin LZ4-20B200; Osram, Germany). Imaging was down the barrel of the FUS transducer and used a Blackfly video camera (BFLY-PGE-13E4M-CS; FLIR Systems, OR, USA) equipped with a 550 nm long-pass dichroic glass filter. A pulsed FUS program was used, setting the transducer to 1.4 MHz, acoustic power up to 18 W, duty cycle 25 %, PRF 0.5 Hz (equivalent to 0.5 s on, 1.5 s off). Processing of the resulting videos concentrated on a small rectangular region-of-interest (ROI) placed just downstream of the FUS focus. After conversion of the video to individual frames, the average ROI pixel intensity was calculated using ImageJ v1.4 (National Institutes of Health, USA) and plotted against frame time. The measured temperatures were then collated and aligned.

### MRI relaxivity

The ability for iTSL to enhance T_1_ relaxation was investigated on two horizontal bore preclinical scanners; a VNMRS (Agilent, Walnut Creek, California, USA) at 7 T, and a BioSpec (Bruker Biospin, Ettlingen, Germany) at 9.4 T. Both used a quadrature volume radio-frequency coil of inner diameter of 39 mm (RAPID Biomedical GmbH, Rimpar, Germany).

Both MRI studies were performed on liquid phantoms with various concentrations of iTSL (with/without DOX) and diluted Gadovist^®^ (a clinically-relevant control). iTSL-DOX and 'released' iTSL-DOX (previously incubated at 45 °C for 3 min) phantoms were used for the 7 T study. T_1_-weighted spin-echo sequence was performed at room temperature with the following parameters: TR 12.06, 20, 30, 40, 50, 60, 80, 100, 120, 150, 180, 200, 250, 300, 350, 400, 450, 500, 700, 850, 1000, 1200, 1500, 1700, 2000, 5000, and 10000 ms; TE 8 ms; average of 2; matrix size 128×128; and FOV 40x40 mm. T_1_ maps were calculated from the relaxometry data by pixel-by-pixel fitting to Equation 1 using ImageJ.


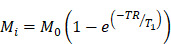
(1)

**Equation 1:** Standard saturation recovery equation used to determine the T_1_ longitudinal relaxation time; where M_i_ is signal intensity, TR repetition time, and M_0_ proton density.

T_1_-relaxometry of iTSL-NO-DOX phantoms at a magnetic field of 9.4 T was carried out in a similar fashion. A fast spin-echo sequence was performed using: TR 45, 50, 60, 80, 100, 120, 150, 180, 200, 350, 450, 500, 750, 850, 900, 1000, 1200, 1500, 1700, 2000, 3000, 5000 and 10000 ms; TE 11 ms; and 2 averages. Sufficient numbers of 2 mm thick coronal imaging slices were used to image volumes of interest with field of view (FOV) 26×26 mm and matrix size 128x128. T_1_ maps were generated from raw data by pixel-by-pixel non-linear fitting to Equation 1 using JIM v8.0 (Xinapse Systems, Alwincle, UK).

In both studies the analysis of images included a selection of ROI drawn by hand and placed on the maps to obtain mean T_1_ relaxation times. The longitudinal relaxivities (r_1_) were calculated from a linear relationship of the relaxation rate as a function of the gadolinium concentrations (Equation 2) using Prism v8.2 (GraphPad Software, San Diego, SA, USA). Gadolinium concentrations from the iTSL used in the phantoms were derived by TXRF quantification as described above.


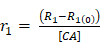
(2)

**Equation 2:** Calculation of molar relaxivity (r_1_); where R_1_ is relaxation rate (equivalent to 1/T_1_), R_1(0)_ is the relaxation rate of the buffer, and [CA] is the contrast agent concentration (in this case Gd^3+^) 24.

### Animal studies

All animal procedures were conducted under the U.K. Home Office regulations and the Guidance for the Operation of Animals (Scientific Procedures) Act (1986). Female 4-6 week old athymic nude mice and CD-1 mice were from Envigo (Huntingdon, UK) and female 4-6 week old Severe Combined Immunodeficiency Hairless Outbred (SHO) mice were from Charles River Laboratories (Wilmington, MA, USA). Unless otherwise stated, injections were given via an *i.v.-*cannula implanted in the tail vein, and used a syringe driver (100 µL/min).

### Gadolinium and doxorubicin pharmacokinetics

Gadolinium clearance study used iTSL (200 µL; [Gd] 1.3 mg/mL) administered to CD-1 mice (n = 6) with blood samples (25 µL each; over 4 h) collected from the caudal vein and transferred into pre-weighed vials along with heparin (2 µL). These were initially kept on ice and then frozen for storage. Portions (10 µL) of each were mixed with a gallium internal standard (10 µL; 4 mg/L) and polyvinyl alcohol aq. (10 µL; 1.2 g/L) before analysis by TXRF as previously described.

In a separate study, CD-1 mice (n = 7) were given an *i.v.* dose of iTSL-DOX (4 mg/kg doxorubicin) and blood samples (55 µL each; over 3 h) collected. These were handled as above but centrifuged (4,500 rpm; 5 min; 4 °C) to isolate plasma before freezing. Doxorubicin extraction was carried out by addition of acetonitrile, centrifugation (10,000 rpm; 5 min; 4 °C) and analysis of the supernatant by LC-MS/MS, using daunorubicin-HCl as an internal standard. This used a Hypersil Gold aQ 50×2.1 mm 3µ reverse phase column (Thermo Scientific; Waltham, MA, U.S.A.) and TSQ Quantum Access mass spectrometer (+ve ESI) with indicative selected reaction monitoring transitions 544.170 → 378.940, 544.170 → 396.950 for doxorubicin or 528.18 → 328.9 for daunorubicin and with calibration against doxorubicin-HCl (20-50,000 ng/mL).

### Tumour induction

MDA-MB-231 cells (6×10^6^ per tumour) were suspended in PBS and then mixed 1:1 (v/v) with Geltrex Matrix (ThermoFisher). The mixture was placed subcutaneously* (s*.*c.)* on the dorsal haunch of each mouse, with either single tumour (right side only) or double tumour (both sides) models prepared according to the study needs. Tumours were considered ready for FUS treatment once they had reached 5-6 mm Ø when measured with a digital calliper.

### Murine FUS

Ultrasound was applied using a Therapy and Imaging Probe System preclinical FUS (TIPS; Philips Research, Netherlands). Mice were anaesthetised with isoflurane/oxygen and placed on a warmed gel pad over an acoustic foam mat to absorb unwanted ultrasound. Two fine-wire thermocouples (T150A; Linton Instrumentation, U.K.) were carefully implanted around the target tumour. Warmed and degassed (by centrifugation) ultrasound gel was used to fill all air gaps between the pad, the mouse, and the TIPS window. Normally the TIPS window was placed 8.0 mm above the tumour surface and FUS was applied at 1.3 MHz, using a 100 % duty cycle. This placed the focus above the skin surface, minimising the risk of skin burns or unwanted tumour ablation but allowing for efficient FUS-induced hyperthermia. Tumour temperatures were measured at 50 ms resolution and TIPS acoustic power settings were adjusted manually (10-20 W) to converge on the target (42/43 °C) and this could then be maintained with little variation. A normal protocol consisted of two rounds of FUS: 20 min pre-injection and 45 min post-injection.

### iTSL-DOX biodistribution

Near-infrared fluorescence (NIRF) and gadolinium quantification was used for imaging the iTSL and to analyse tumour content. Mice bearing single tumours were anesthetised and iTSL (200 μL; [Gd]: 0.56 mg/mL) administered *i.v.* via the tail vein by slow infusion (35 μL/min). The FUS group received two rounds of FUS (20 min pre-inject, 43 °C, 3 min; 45 min post-inject, 42 °C, 3 min) while the control received no FUS. NIRF imaging used a Maestro EX (Perkin Elmer, MA, USA) with EX 704 nm, EM 740-950 nm, 20 nm slicing, and unmixed against CF750-DSA. At 4 h the animals were sacrificed and tumours excised for gadolinium analysis by TXRF.

### Anti-tumour efficacy

Two separate experiments were performed in single and double tumour mouse models. The single tumour study used three groups, all given injections *i.v.* tail vein: (i) control (n = 5) which received only PBS; (ii) doxorubicin-HCL aq. (n = 5) at 4 mg/kg; and (iii) iTSL-DOX (n = 9) at equivalent to 4 mg/kg doxorubicin and two FUS treatments (Figure S3). NIRF imaging was used to track distribution and tumour uptake of the iTSL-DOX for 24 h post-injection. Tumour size and mouse body weights were recorded until they reached pre-determined endpoints (>10 mm longest axis or >20 % loss of pre-study weight) whereupon the animal was sacrificed. Tumour volume calculations were obtained using (*d*^2^ × *D*)/2 where d is the shortest tumour axis and D is the longest.

The double tumour study used two groups: (i) no-drug reference (n = 3) received only FUS on the right-side tumour, leaving the left to grow normally as a no-FUS control; and (ii) iTSL-DOX (n = 10) at equivalent to 6 mg/kg doxorubicin and again FUS only on the right-side. Injections were given *i.v.* tail vein and FUS followed the same protocol. Tumour size and mouse body weights were recorded as before, and NIRF tracking of the iTSL was continued for 2 weeks post-treatment.

*In vivo* apoptosis assessment was carried out using the same double tumour model (n = 3) treated with iTSL-DOX but omitting CF750-DSA. *Annexin-Vivo* 750 (100 µL; Perkin Elmer) was administered 48 h after treatment and apoptosis assessed by NIRF imaging after 4 h, according to the manufacturer's protocol. Tumours were then excised and fixed for H&E staining.

### Murine tumour MRI

For MRI, tumours were established and grown to 5-6 mm Ø as previously described (n = 3). Mice were anesthetised and injected *i.v.* via the tail vein with iTSL (200 μL; [Gd] 1.37 mg/mL). MR imaging was performed beforehand and at 1 h, 3 h and 5 h afterwards. Animals were placed in the radiofrequency coil and flanked with a vial containing Gadovist® ([Gd] 3 ± 0.01 mg/L) as a reference before placement into the magnet bore and maintenance at 37 °C. T_1_-relaxometry and typical T_1_-weighted images were performed at 9.4 T. T_1_-relaxometry was performed using a fast spin-echo sequence with the following parameters: TR 240, 400, 600, 800, 1000, 1200, 1500, and 3000 ms; TE 11 ms; matrix size 128×128; 1 scan; FOV 35×35 mm. Eight contiguous transverse 0.7 mm thick slices were collected, covering the tumour. Typical higher signal-to-noise T_1-_weighted images were also obtained with the same sequence at TR 300 ms; TE 7.765 ms; 2 averages; FOV 35×35 mm, and matrix size 175×175.

T_1_ maps were generated as described above and matched ROI were identified in each slice for tumour, muscle (non-tumour control), and Gadovist® (positive control) in the adjacent-placed tube. Collated pixel intensities from these areas were combined for each animal and underwent frequency distribution analysis. To the resulting histograms, non-linear regression analysis was applied fitting to a Gaussian curve and the resulting best-fit mean and SD values cross-compared for each animal (n = 3), time-point, and ROI. After ANOVA (1-way) significance analysis, these values were then combined across the subgroups to give overall distribution per subgroup. All statistical analysis was performed using Prism v 8.2.1 (Graphpad Software, San Diego CA, USA).

### Shelf-life stability

The effects of short- and long-term storage on doxorubicin release were assessed using iTSL-DOX kept in a sterile vial at ~5 °C. The short-term study used samples warmed to room temperature for 10 min, 3 h, or 24 h before incubation (32-46 °C; 3 min) and fluorescence analysis. The long term study used samples taken directly from a 5 °C storage vial over 3 months and analysed for doxorubicin release, particle size, and polydispersity index.

## Results & Discussion

### Lipid synthesis and preparation of iTSL-DOX

**Scheme 1:**
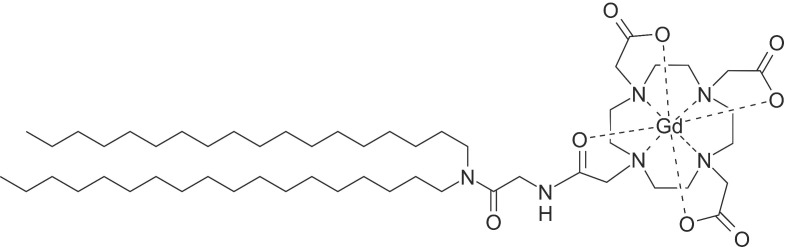
** Chemical structure** of Gd.DOTA.DSA, a lipidic magnetic resonance imaging contrast agent.

Two imaging-lipids were used in this study. Gd.DOTA**.**DSA (Scheme 1) is an MR contrast agent synthesised and characterised as previously described (Supplementary Material; Scheme S1) 21,25. DOTA-chelation was selected due to the reported efficiency of Gd^3+^ incorporation and high stability of the resulting complex. This is reported to significantly reduce transmetalation risk compared to classical diethylenetriamine pentaacetate derivatives 26. After purification by liquid chromatography Gd.DOTA.DSA provided a ~95 mol% gadolinium loading as assessed using TXRF.

iTSL were prepared with a Gd.DOTA.DSA composition of 30 mol% (of total lipid) and underwent an iterative series of formulation optimisations to derive required physical and thermal release characteristics, while retaining a high labelling % for effective MR imaging. A second NIRF label (CF750.DSA) was included at <0.1 mol% and its presence did not appear to modify iTSL characteristics (save for the expected fluorescence). Doxorubicin loading was by incubation near the lipid membrane phase transition temperature, then purification by size exclusion chromatography. Each batch was characterised by DLS, DSC, HPLC, and TXRF with representative values: Ø Z_avg_ 179 ± 3 nm, PDI 0.2 ± 0.01; *ζ* -3.2 ± 0.1 mV; [Gd] 560 ± 16 µg/mL; [doxorubicin] 690 ± 15 µg/mL; drug/lipid ratio of 0.03; T_m_ 43.5 °C. These were considered in-line with our expectations, judging from previous experiences and reports from the literature.

### Thermally-induced doxorubicin release

**Figure 1:**
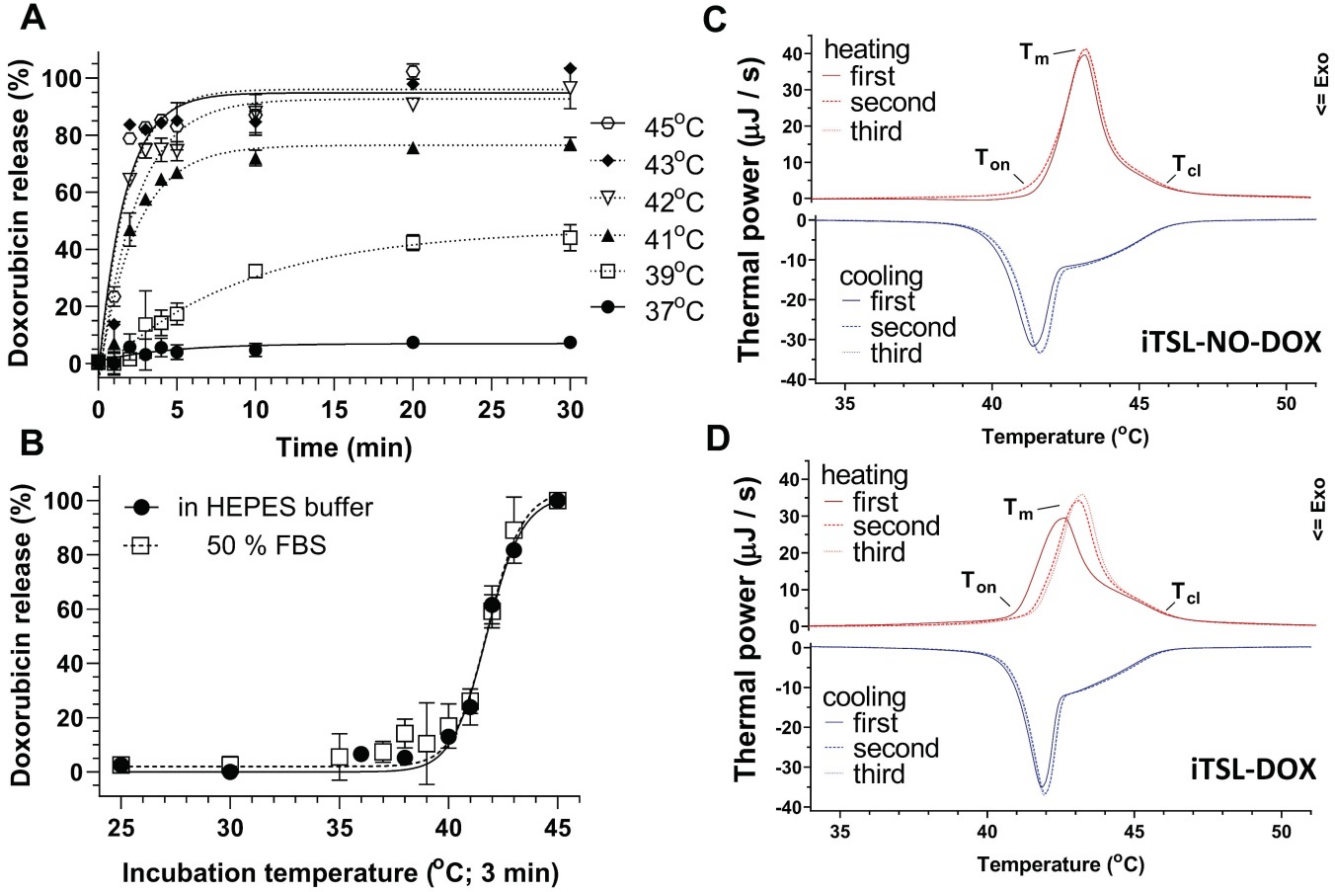
** iTSL-DOX characterisation**: **(A)** Doxorubicin release from iTSL at various temperatures in HEPES; **(B)** for 3 min at various temperatures in comparison with buffer containing 50 % (v/v) fetal bovine serum (FBS). Release is monitored by the increase of intrinsic doxorubicin fluorescence (Ex_480_/Em_590_ nm) as it leaves the self-quenched encapsulated state (n = 3; mean ± SD);** (C)** Liquid-phase differential scanning calorimetry of iTSL without and; **(D)** with encapsulated doxorubicin. Each study consisted of 3x sequential heating-cooling rounds from 25-70 °C at 1 °C/min. iTSL were in HEPES/glucose buffer and used the same as a thermal reference. Indicative onset (T_on_), melting (T_m_), and closure temperatures (T_cl_) for each heating thermograph are: iTSL-NO-DOX: (first) T_on_ 41.2, T_m_ 43.3, T_cl_ 45.8, (second) T_on_ 41.1, T_m_ 43.3, T_cl_ 46.0; iTSL-DOX: (first) T_on_ 40.3, T_m_ 42.7, T_cl_ 46.0, (second) T_on_ 40.9, T_m_ 43.2, T_cl_ 46.0; all ± 0.2 °C. T_on/cl_ were calculated as the first and last temperatures at which the thermal power was 5 % of T_m_.

An effective TSL-drug needs to retain its cargo under normal *in vivo* conditions but rapidly release it on application of mild hyperthermia (~42 °C). Encapsulated doxorubicin has strongly self-quenched intrinsic fluorescence; this allows release from iTSL-DOX to be assessed by monitoring the intensity increase after incubation over a range of temperatures and times. Figure 1A indicates little or no drug release on incubation at 37 °C for 30 min in HEPES/glucose buffer. This was followed by a sudden increase of fluorescence intensity at temperatures ≥41 °C, with more than 80 % of the encapsulated drug released in 2 min at 43 °C. iTSL-DOX demonstrated almost identical release profiles in buffer and serum-like conditions (Figure 1B) which bodes well for its use *in vivo* 27.

This thermally-induced drug release aligns well with liquid-phase DSC thermograms collected for iTSL-NO-DOX (without loaded doxorubicin) and iTSL-DOX (Figure 1C-D). DSC investigates the changes in bulk heat capacity on heating or cooling from 25-70 °C (1 °C/min; 3× sequential heat-cool cycles). The resulting peaks/troughs are mainly due to temperature-driven phase changes in the lipid membrane; these are associated with pore formation and release of the encapsulated drug. iTSL-NO-DOX and iTSL-DOX have slightly different first heating thermograms, with the latter showing a 1 °C down-shift in the peak leading edge to ~41.5 °C (Figure 1D). This initiation temperature matches the doxorubicin release point seen from the fluorescence studies. This strongly suggests that DSC is able to detect the release of encapsulated doxorubicin during this first round of heating. Subsequent cycles are then the same as iTSL-NO-DOX, likely because the drug release is complete. Overall, doxorubicin release parameters suggest that iTSL-DOX formulation has been successfully optimised to adjust for the high mol% of MRI CA lipid 28,29.

TSL tend to be unstable when incubated in serum 30 and this has previously caused difficulties during clinical development. For example, a widely used LTSL (lysolipid-based temperature sensitive liposome) formulation released 50% doxorubicin after 30 min in 20 v% serum 31. This was attributed to the presence of adhered plasma proteins causing ~70% of the lysolipid to dissociate from the liposomal membrane within the first hour. This likely resulted in significant drug leakage in the blood stream 32,33. We investigated doxorubicin release from iTSL-DOX incubated at 37^o^C in buffer and in 50 v% foetal bovine serum. After 60 min incubation there was minimum leakage (~5 %) doxorubicin into buffer but 30% into serum (Figure S4). This was a significant improvement over previously reported LTSL and suggested that doxorubicin leakage in blood circulation should be greatly reduced.

### FUS-induced doxorubicin release

**Figure 2:**
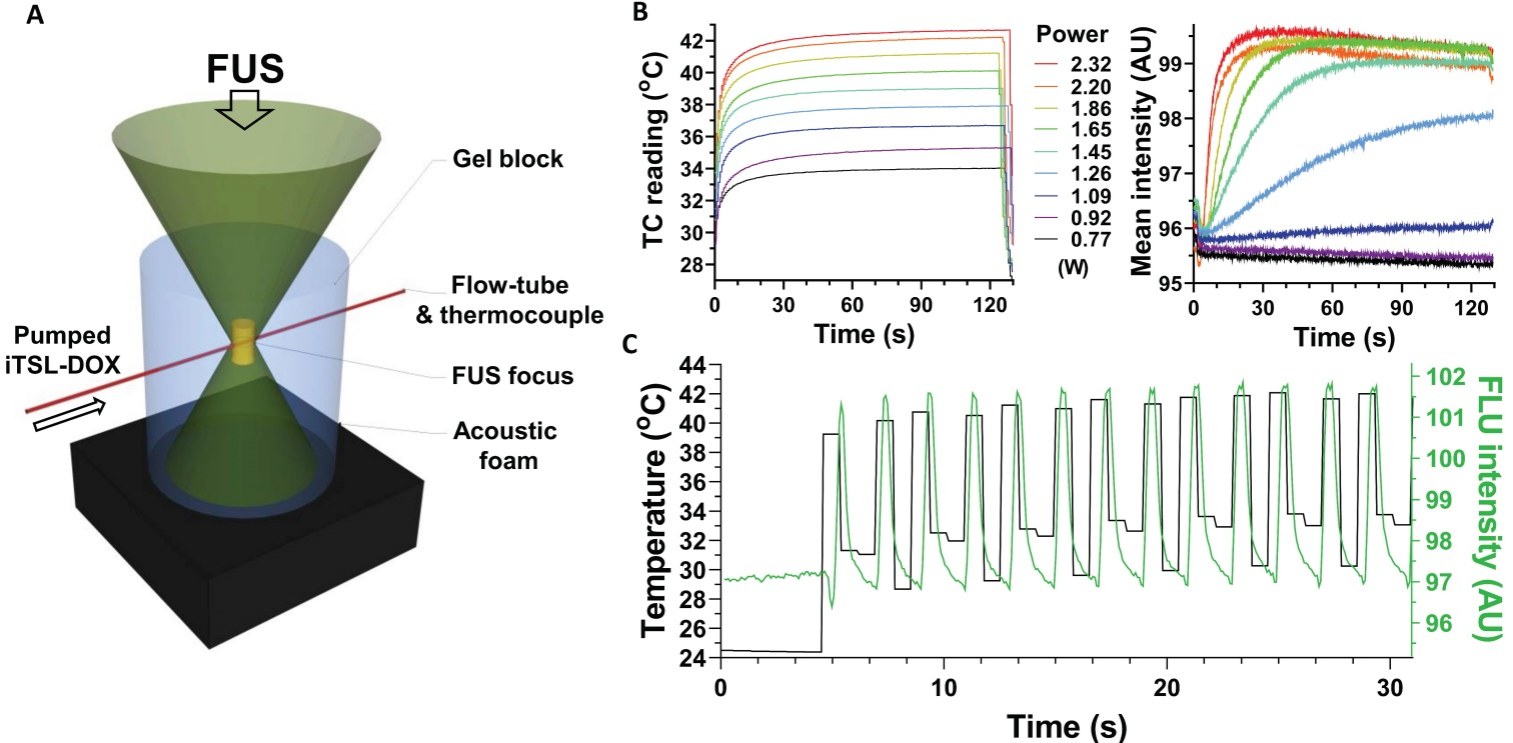
** Assessing doxorubicin release under FUS,** measured by intrinsic doxorubicin fluorescence; **(A)** schematic showing a polyacrylamide gel embedded flow-tube below a focused ultrasound transducer (Figure S1); **(B)** graphs of thermocouple (left) and fluorescence intensity (right) readings seen with increasing power levels of constantly applied FUS, using a fresh bolus of iTSL-DOX for each 2 min insonation; **(C)** Fluorescence intensity and temperature plotted against time under pulsed FUS and constant iTSL-DOX flow.

Incubation studies at different temperatures were a facile method to assess iTSL-DOX thermossensitivity but did not demonstrate drug release under FUS insonation. Here, we investigated FUS-induced doxorubicin release in real time using a custom made flow-tube setup (Figure 2A). A solution of iTSL-DOX was pumped through a capillary tube embedded in a block of tissue mimicking gel 34. FUS was applied using a small-animal transducer array and doxorubicin release was monitored using fluorescence imaging with a video camera, while a thermocouple tracked the temperature near the FUS focus (Figure 2, Figure S1).

Initial studies used the tube as a refillable sample cell, applying a particular degree of FUS acoustic power for 2 min, then washing out, replacing with a fresh aliquot of iTSL-DOX, and repeating at a higher power. As expected, the focus peak temperature increased directly with applied FUS power (Figure 2B left) as did the doxorubicin fluorescence (Figure 2B right). The latter also showed release profiles similar to those from the thermal-induced release studies, with doxorubicin release rates dependent on the acoustic power applied.

To investigate the doxorubicin release responsiveness, the setup was adjusted to have a constant flow of fresh iTSL-DOX through the tube (modelling blood capillaries) while applying pulsed FUS at a power level sufficient to rapidly raise the focus temperature to our expected *in vivo* target of ~41 °C. Doxorubicin fluorescence then showed a rapid and temporally coherent increase that matched the measured temperature spikes (Figure 2C). Acoustic power is an important parameter that can control the rate of the release and (with this system) at 2.32 W the drug release appears effectively instantaneous. This implies that spatiotemporal control of the application of the acoustic power can lead to precise remote control of the drug released.

### *In vitro* MR relaxivity

MR contrast agents (CA) are used to increase the imaging contrast between tissues, usually by shortening the longitudinal (T_1_) relaxation time or the transverse (T_2_) relaxation time. There are two main approaches to developing liposomes that enhance tumour MR T_1_ contrast: (i) encapsulation of small molecule Gd^3+^-chelates into the aqueous core, which then label the tumour after thermal release; or (ii) the attachment of anchored Gd^3+^-chelates to the lipid membrane. The latter allows for improved water accessibility and better contrast since the interaction of Gd^3+^ with the surrounding water protons is not inhibited by the lipid membrane 35.

**Figure 3:**
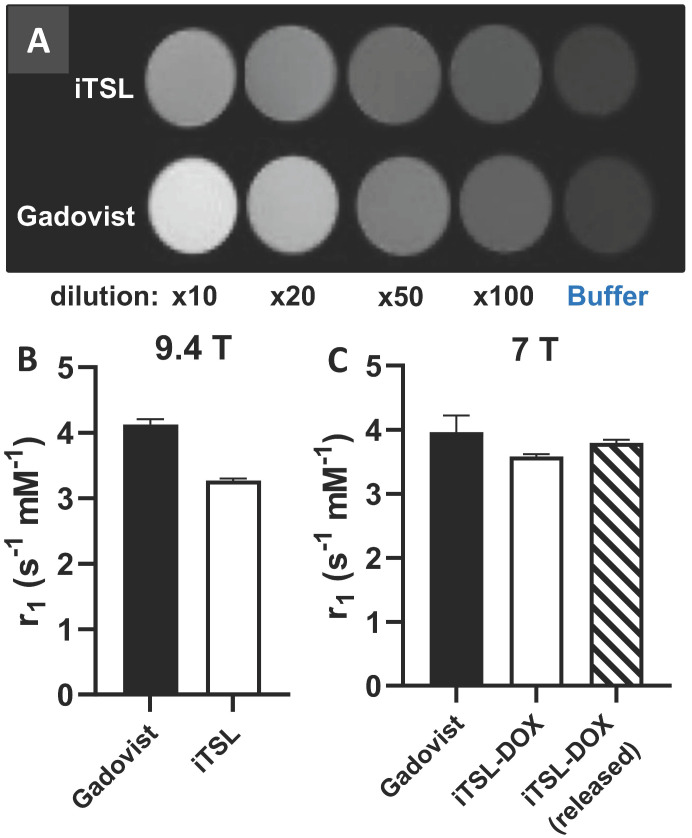
** MRI T_1_ relaxivity measurement of iTSL and Gadovist®** (positive control) in phantoms; **(A)** 9.4 T MRI images of samples with approximately matched gadolinium content (initially 1.46 mg/mL for Gadovist®, 1.36 mg/mL for iTSL) using HEPES/glucose buffer as a baseline and for dilution; **(B)** Molar relaxivities (r_1_) were calculated from the relaxation rate change as a function of gadolinium concentration (as measured by TXRF). A higher r_1_ implies a stronger contrast agent; **(C)** Equivalent r_1_ values for doxorubicin loaded iTSL using 7 T MRI, with initial gadolinium concentrations: 0.43 mg/mL Gadovist®; 0.26 mg/mL iTSL and again using HEPES/glucose buffer for dilution. The iTSL-DOX (released) sample was previously incubated for 3 min at 45 °C to thermally activate the liposomes.

Figure 3A presents T_1_-weighted images of phantoms containing different dilutions of iTSL and Gadovist® (a positive control) at comparable molar concentrations of Gd^3+^ and at a magnetic field strength of 9.4 T. iTSL showed enhanced signal intensity dependent on the concentration, similar to Gadovist® serial dilutions. Figure 3B-C shows the molar T_1_ relaxivities of iTSL and Gadovist® at 9.4 and 7 T. This is a measure of the efficacy of the CA to decrease the T_1_ relaxation time of water protons in its surrounding. The greater the r_1_, the smaller the amount of the CA is needed to achieve the same T_1_ decrease and hence brightness enhancement in the T_1_-weighted MR image 36. At 9.4 T iTSL shows lower efficiency as a T_1_-CA compared to the Gadovist® - one of the most efficient small-molecule cyclic-gadolinium CA used in clinic, however magnet field strength affects relaxivity of contrast agents 37. The relaxivity of Gd-based liposomal CA is generally higher when the common clinical magnets (1.5 and 3T) are used. At 7 T, the molar relaxivities of iTSL-DOX and Gadovist® were similar and pre-heating to release the doxorubicin gave a slight improvement. We judge this is likely due to a change of water permeability across the iTSL membrane. Increased membrane porosity improves water accessibility to the liposome core, allowing the normally isolated inner surface Gd.DOTA.DSA to have a contrast enhancing effect. Under either condition, iTSL appeared to be an effective T_1_-CA.

### Shelf-life stability

There are only a few published studies that investigate how long-term storage affects TSL characteristics. TSL formulations contain lyso-lipids and may show limited colloidal or drug-retention stability (even at reduced temperature). This can be a particular issue at lab scale production where the methods of preparation, sterility assurance, and storage conditions are likely to be sub-optimal.

To inform on this, we introduced a stability study to investigate the effect of short- and long-term storage on the integrity of aseptically produced iTSL-DOX (Figure S5). Samples were initially left at room temperature for 3 or 24 h - durations selected to mimic the time range that a formulation might be left on a bench during a human trial or large animal study. iTSL-DOX showed only minimal changes to the doxorubicin release profile after 24 h and none at 3 h, indicating a robust formulation that retains its properties for a reasonable time outside of a fridge. Long-term stability was then assessed using samples taken at intervals from iTSL-DOX in cold-storage (~5 °C) for up to 3 months. Again there was no apparent change to the release profile as the liposome ages, confirming the formulation robustness. DLS analysis of the same samples also showed no significant changes to average nanoparticle diameter or the PDI. We conclude that iTSL-DOX has good storage robustness, which obviates the requirement for fresh preparations between rounds of *in vivo* studies.

### Avoidance of gadolinium toxicity

Free Gd^3+^ cations are toxic due to interference with Ca^2+^-dependant biochemical processes, amongst other effects 38. This toxicity is greatly reduced when the metal is chelated but there are currently concerns due to the demonstrated links between the use of Gd-based MRI contrast agents and rare but serious incidents of nephrogenic systemic fibrosis in patients with poor kidney function 39. Therefore, there is a question as to the safety of gadolinium contrast agents that may 'leak' Gd^3+^ due to chelation competition from biomolecules in the body 40.

We investigated the integrity of iTSL's Gd^3+^ chelation under challenging conditions. Figure S6 shows a log plot of the leakage of Gd^3+^ (as assessed by TXRF) through dialysis membranes against water or serum, over two days. This is compared to the behaviour of matched concentration controls of unchelated Gd^3+^ aq. from gadolinium standards. The pore size of the dialysis cassette (10 kD MWCO) blocked intact iTSL but allowed the passage of 'free' (or small molecule bound) Gd^3+^. Hence gadolinium assayed from the external chamber was either from the control or was initially part of Gd.DOTA.DSA and subsequently extracted by competition from the medium. FBS was assessed as serum contains many of the proteins and ions found in blood and provides a fairly realistic approximation of the conditions that Gd.DOTA.DSA would encounter once injected *i.v.*

It was observed that while the 'free' Gd^3+^ controls diffusion equilibrated, there was very little metal detected from the iTSL and no obvious increasing trend over the 48 h study. This is in-line with previous reports which demonstrated greatly reduced Gd^3+^ leakage from the macrocyclic chelators under serum conditions compared to linear chelators 38,39. We concluded that iTSL was safe for *i.v.* administration and further clinical development.

### Blood clearance profiles in mice

**Figure 4:**
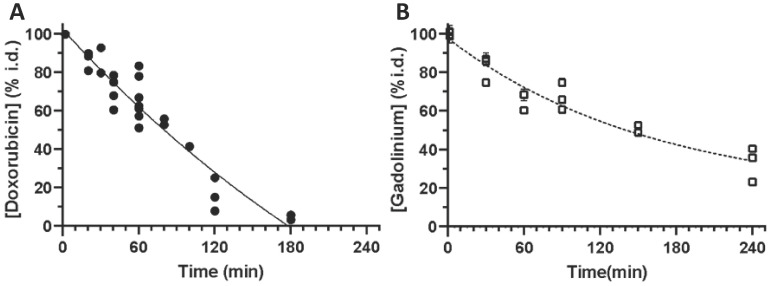
** Clearance of iTSL-DOX; (A)** doxorubicin (n = 7) and; **(B)** gadolinium (n = 6) from mouse blood circulation, shown as % of injected dose. Mice were injected (*i.v.* tail) with iTSL-DOX (4 mg/kg equivalent doxorubicin), then blood samples were collected at time points. Values are the mean of 2-3 repeat analyses per sample ± SEM.

To develop a better understanding of the pharmacokinetic behaviour of iTSL-DOX, we used LC-MS/MS and TXRF analysis to assess the blood clearance rates of doxorubicin and Gd.DOTA.DSA (Figure 4). The iTSL-DOX was injected *i.v.* and blood samples were taken at predetermined time points. Doxorubicin (whether encapsulated or not) appears to be cleared from the blood stream by 3 h, with a t_1/2_ of ~80 min. This assay detected both iTSL encapsulated doxorubicin and 'free' drug. In comparison with previously presented data on similar TSL-DOX formulations, ours indicate a slightly shorter half-life but are in general agreement with the short term kinetics of thermosensitive liposomes in blood. Blood circulation stability is a concern, as previously reported TSL-DOX has demonstrated a tendency to leak doxorubicin - a factor that may have significantly limited its clinical success. We also measured the liposome kinetics by assessing blood gadolinium levels. It is evident that while doxorubicin circulates in blood up to 3 h post-administration, Gd.DOTA.DSA is detectable for much longer. Considering the slow doxorubicin leakage under blood-like conditions (Figure S4) it seems reasonable that past 3 h some iTSL are still circulating but are empty of doxorubicin and Gd.DOTA.DSA eliminates from the blood with a t_1/2_ of ~ 150 min.

TSL formulations are generally designed to avoid the very long blood circulation times usually observed with liposomes like Doxil®. They are intended to be combined with hyperthermia and to efficiently deliver a drug into the tumour interstitial and/or intravascular space. To achieve this regional delivery TSL should be activated while they circulate at high concentrations and while they retain their drug cargo. This indicates that the triggered drug release should occur before the normal blood clearance t_1/2_ time point of the drug delivered by TSL. This is the suggested time window for maximal tumour drug concentration once a thermal trigger is applied 32. Shorter half-lives can have the additional advantage of diminishing exposure of off-target tissues to doxorubicin. This may have a significant advantage of reducing doxorubicin-related cardiotoxicity, as reported for Doxil® 43. But at this point there are no studies showing a comparison of the therapeutic index between Doxil® and TSL-DOX.

### T_1_-weighted MRI of iTSL in tumour bearing mice

**Figure 5:**
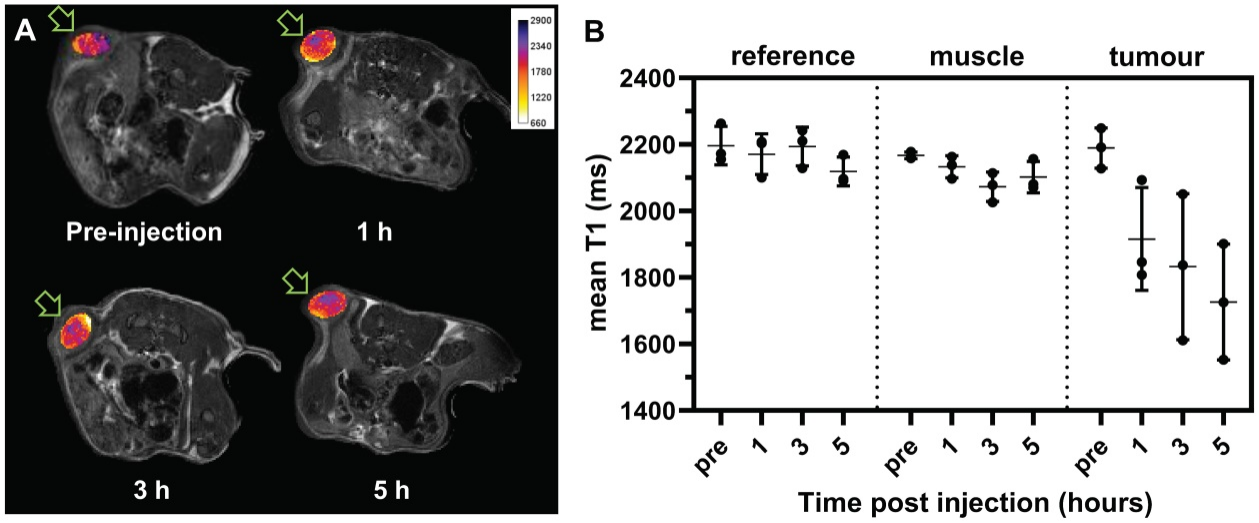
** Spin-echo transverse T_1_ weighted MR imaging,** iTSL (1.37 mg/mL gadolinium) was injected (*i.v.* tail vein) to mice, **(A)** Imaging (9.4 T) of the same representative animal pre-injection, then 1 h, 3 h, and 5 h post-injection. T_1_-weighted tumours images are overlaid with T_1_ relaxivity maps (in false colour) for the region-of-interest (ROI), as indicated with arrows; **(B)** Comparative T_1_ values for selected volumes-of-interest (combining the ROIs from matched slices) from a single-tumour mouse study (n = 3; individual values •, mean as horizontal bar, SD as vertical bar); see also Figure S7. A lower T_1_ implies a stronger contrast enhancement.

While MRI phantoms show that iTSL has good T_1_-relaxivity, it is essential to demonstrate that they can also be detected *in vivo*. Figure 5A shows typical T_1_-weighted images of subcutaneously implanted tumours in mice before and after iTSL administration**.** Similar studies reported by Liu *et al.* used the relative signal intensity from T_1_-weighted images of kidney and liver to study the kinetics of Gd.DTPA labelled liposomes 44. In our experiment, *i.v.* injection of iTSL resulted in an increased MR signal intensity in the tumour within an hour. Figure 5B shows the changes in mean T_1_ of tumour volumes-of-interest (combining ROI across multiple imaging slices) compared to a reference vial of Gadovist^®^ CA and a reference tissue (skeletal muscle). The T_1_ of reference regions were not expected to change significantly over time but tumours showed decreased T_1_ due to the accumulation of iTSL and this continued until the end of the study. An increase in the variability of the T_1_ values across the tumour were also observed, which was attributed to high tissue heterogeneity (due to the developing necrotic core) and differential iTSL uptake depends on the amount of blood perfusion. A histogram analysis approach was used to better distinguish the changes under these heterogeneous conditions (Figure S7).

The observed reductions in mean T_1_ demonstrated the accumulation of iTSL in the tumour soon after injection and its persistence for at least 5 h post-injection. This finding supports the hypothesis that iTSL can be tracked post-injection for spatiotemporal control of the FUS triggered release. For a radiologist working with MRgFUS a change in the MR signal from the tumour could indicate the optimal time for application of FUS. It could also confirm that the treatment was successful, as there would likely to be an enhanced tumour signal post treatment.

### Design of the FUS protocol

iTSL-DOX pharmacokinetics information directs the choice of FUS parameters, in particular the timing respective to liposome administration. The blood clearance results showed that the remaining iTSL-encapsulated doxorubicin is likely to be significantly reduced after 1 h and effectively eliminated after 3 h. This suggests that FUS should be applied soon after injection to best affect doxorubicin release from iTSL that are still loaded with drug. However, this needs to be balanced by allowing sufficient time for iTSL-DOX to accumulate into the tumour. The use of multiple rounds of FUS could trigger release from iTSL-DOX while still being infused, similar to the release observed in the *in vitro* system in Figure 2. Our previous imaging results with a similar iTSL-topotecan formulation demonstrated that the drug is rapidly released on application of FUS and that treatment repetition can maximise the available drug in the tumour 21. Short rounds of FUS were also found to be equally efficient compared to prolonged continuous hyperthermia in a recent study 45. We concluded that brief (≤5 min) periods of FUS hyperthermia (≤43 °C) would be sufficient to induce drug release (intravascular and/or interstitial). In our experience brief FUS treatments are also safer than longer ones*,* being less stressful for the animals, reducing the risk of skin/tumour burns or other over-heating related damage, and (importantly) avoiding false positive results (e.g. from hyperthermic ablation, leading to tumour tissue damage masking the effect of the released chemotherapeutic).

Application of FUS before drug injection has been reported to improve therapeutic efficacy. For example, ThermoDox® preclinical development information strongly suggests that the heating of the tumour needs to precede injection of the TSL 8. Other studies have demonstrated effective treatment with FUS applied just before and then during infusion 46, or immediately after injection 47. In addition, the phase-III HEAT clinical trial computer modelling led to application of radiofrequency ablation 15 min post-infusion 48 and the TARDOX study indicated application of FUS shortly after infusion 20. The high variability of TSL FUS protocols across the literature suggests that better feedback tools are required to allow the identification of optimum timing. Feedback through imaging of the TSL during treatment can make the process easier for the radiologists. For instance, FUS could be applied once MRI contrast has reached a predefined level (contrast) in the tumour. Under clinical conditions FUS-induced hyperthermia would be applied and controlled using concurrent imaging (MRgFUS) which should identify the tumour and margins but would also be used to monitor the tissue temperature, iTSL accumulation and provide a confirmation that appropriate dose has reached the tumour 49.

### NIRF imaging of iTSL+FUS treated mice

Biodistribution studies were carried out by administering NIRF-labelled iTSL-DOX into mice with human TNBC (MDA-MB-231) cell tumours. These were placed on the haunch to allow easier and safer access of the FUS beam and were ~5 mm diameter on treatment day. Two groups received iTSL-DOX but only the one underwent FUS-induced hyperthermia. Here we combined one brief sonication before injection with a second round after but with enough delay to allow enhanced iTSL uptake into the tumour. NIRF tracking of iTSL uptake allowed us to select a 45 min post-injection time point for the second FUS, leading to significant tumour accumulation while still corresponding to ~70% availability of injected dose of doxorubicin in the blood stream (Figure 4). At this point it is likely that iTSL-DOX still encapsulates the great majority of the doxorubicin since both doxorubicin and gadolinium are found at similar % of injected dose.

**Figure 6:**
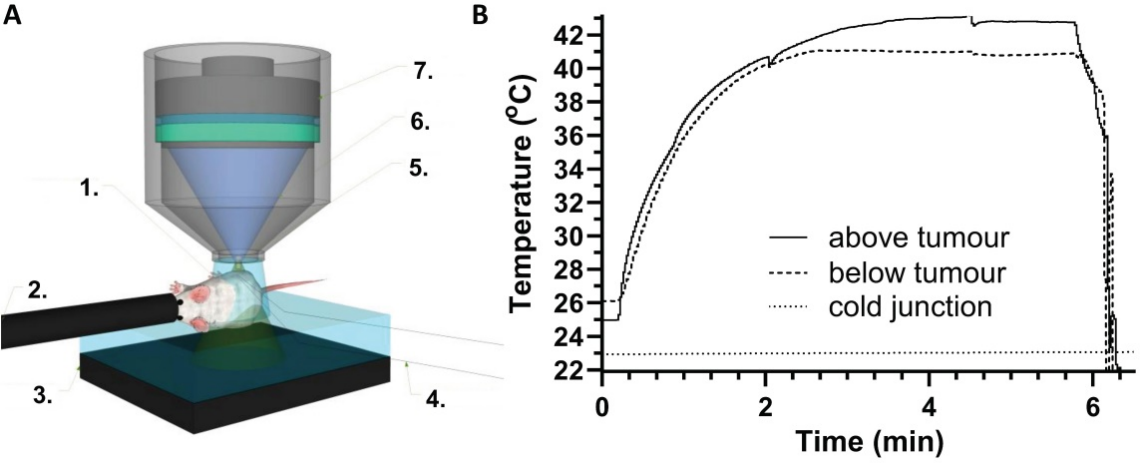
** Preclinical FUS studies. (A)** Mouse focused ultrasound showing key components: 1. degassed gel; 2. anaesthetic; 3. acoustic foam; 4. thermocouples; 5. focus; 6. ultrasound biconic; 7. transducer;** (B)** Tumour temperatures measured using fine-wire thermocouples implanted *s.c.* above and below the tumour (with respect to the transducer focus location). The cold junction is in the recording electronics and ~ 2 ^o^C above RT.

In all experiments the FUS focal volume was deliberately placed slightly above and outside the targeted tumour. This was done to avoid the formation of a 'hot spot' within the tumour body, ensuring the effects seen were due to FUS-induced mild hyperthermia and not ablative tissue damage. To control the applied FUS power, tumour temperatures were monitored using fine-wire thermocouples that were implanted around the tumour (Figure 6). This was to substitute for the more elaborate MR thermal maps provided by clinical MR-guided FUS equipment 50. Thermocouples were inserted below and above the tumour, avoiding the FUS focal volume, and temperatures were monitored throughout the treatment. Figure 6B provides typical thermographs achieved during an FUS treatment showing that temperatures reach a constant plateau.

**Figure 7:**
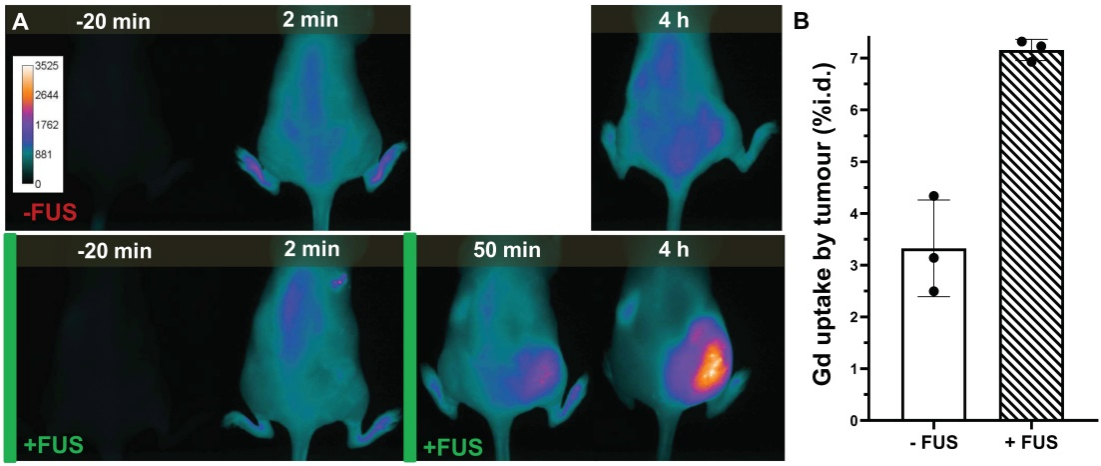
** Representative imaging of iTSL-DOX uptake; (A)** Near-infrared fluorescence imaging comparison of mice injected (*i.v.* tail; t = 0) with iTSL-DOX, and without (-FUS) or with (+FUS) focused ultrasound on the right side. Images are shown pre-, then post-injection and the FUS treatments are indicated by the green bars. The CF750-DSA liposome label was excited at 704 nm and fluorescence emission collected over 740-950 nm. **(B)** Gadolinium tumour concentration (n = 3; mean ± SD; normalised by tumour mass) assessed by TXRF.

NIRF imaging allows monitoring of the level of iTSL within the tumour compared to the rest of the body 46. We selected it as a semi-quantitative but robust method that was expected to significantly facilitate clinical translation 51. Figure 7 shows representative mice from groups that have been administered iTSL-DOX either with or without FUS and then undergone real-time tracking by NIRF imaging. It is evident that the absence of FUS led to little accumulation of iTSL in the tumour. In contrast, brief FUS treatments caused a substantial increase in tumour-localised signal. We monitored mice up to 4 h, at which point the remnant circulating iTSL are likely to be empty and the rate of their partition in the tumour compartment should be decreasing 51. We also TXRF analysed tumour samples for gadolinium content at 4 h (Figure 7B). Given the chelation stability of Gd.DOTA.DSA this should be a reasonable indicator for the amount of iTSL that has been retained in tumours at this time point. At 4h post injection, FUS-treated tumours levels were more than double that of the untreated group.

### Doxorubicin release and iTSL clearance

**Figure 8:**
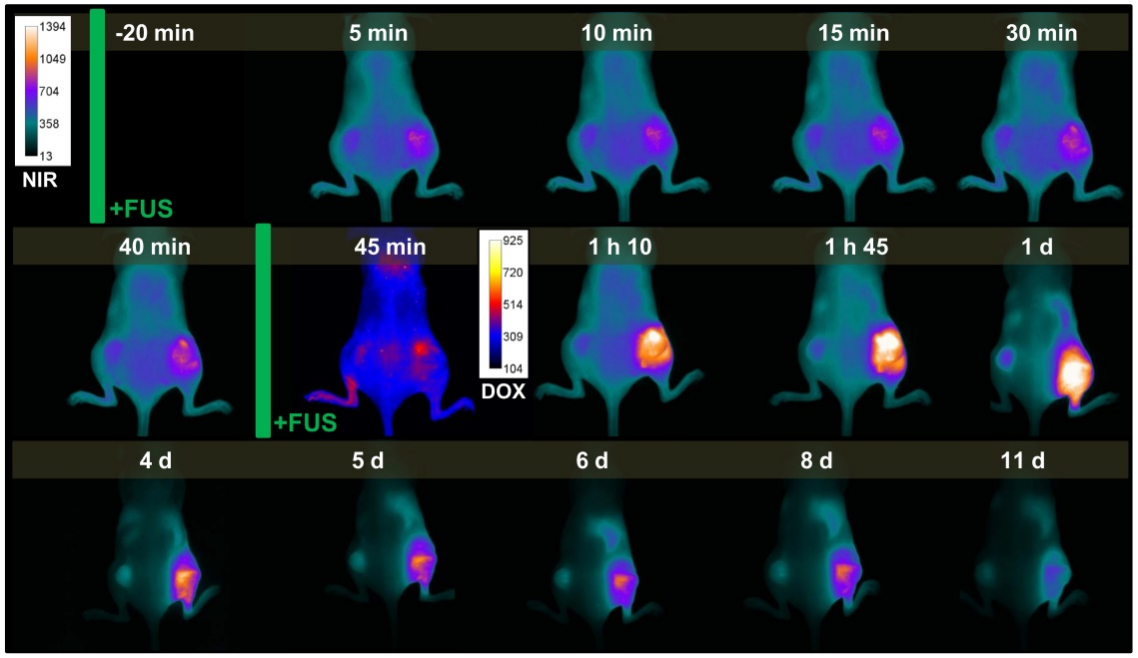
** Representative example of near-infrared (NIRF) and doxorubicin fluorescence imaging** during the dual-tumour study**.** Time points are pre/post-injection and focused ultrasound (FUS) treatments are indicated by green bars. Right tumour received FUS, while left tumour did not receive FUS. NIRF images are coloured cyan-yellow, doxorubicin are indigo-red.

We also assessed iTSL-DOX + FUS treatment on animals bearing two tumours (one on each side). In this double-tumour model, the left side received no FUS whilst the right side was treated as before. This allows FUS effects to be assessed more directly as each animal also acts as control. Figure 8 presents real-time NIRF imaging of iTSL-DOX kinetics on a mouse bearing double MDA-MB-231 tumours and with the optimised FUS protocol applied on the right-hand side. It is evident that FUS-treated tumour allowed accumulation to a much larger extent compared to the FUS-untreated tumour. It is also evident that intrinsic doxorubicin fluorescence (shown here in red against blue) is distinguishable in the +FUS tumour to larger extent compared to the untreated and only when the second round of hyperthermia was applied.

Figure 8 also shows that the CF750.DSA lipid appears to clear slowly from the treated tumour for up to 2 weeks post-administration. The signal from the untreated tumour appears to fade more rapidly. It is possible that FUS enhances the permeation and incorporation of liposome components in the tumour. The concept of FUS-enhanced delivery of lipophilic drugs transferred this way has not yet been explored.

### Efficacy of iTSL-DOX + FUS

Once the FUS protocol had been demonstrated to be effective at increasing iTSL-DOX uptake, we expanded to an efficacy study using a single (right-side) TNBC tumour model and groups: (i) nil treatment; (ii) doxorubicin alone; and (iii) iTSL-DOX with FUS. Doxorubicin dosages were matched at 4 mg/kg and the FUS, imaging, and size/weight measurement timings were as described in Figure S3.

**Figure 9:**
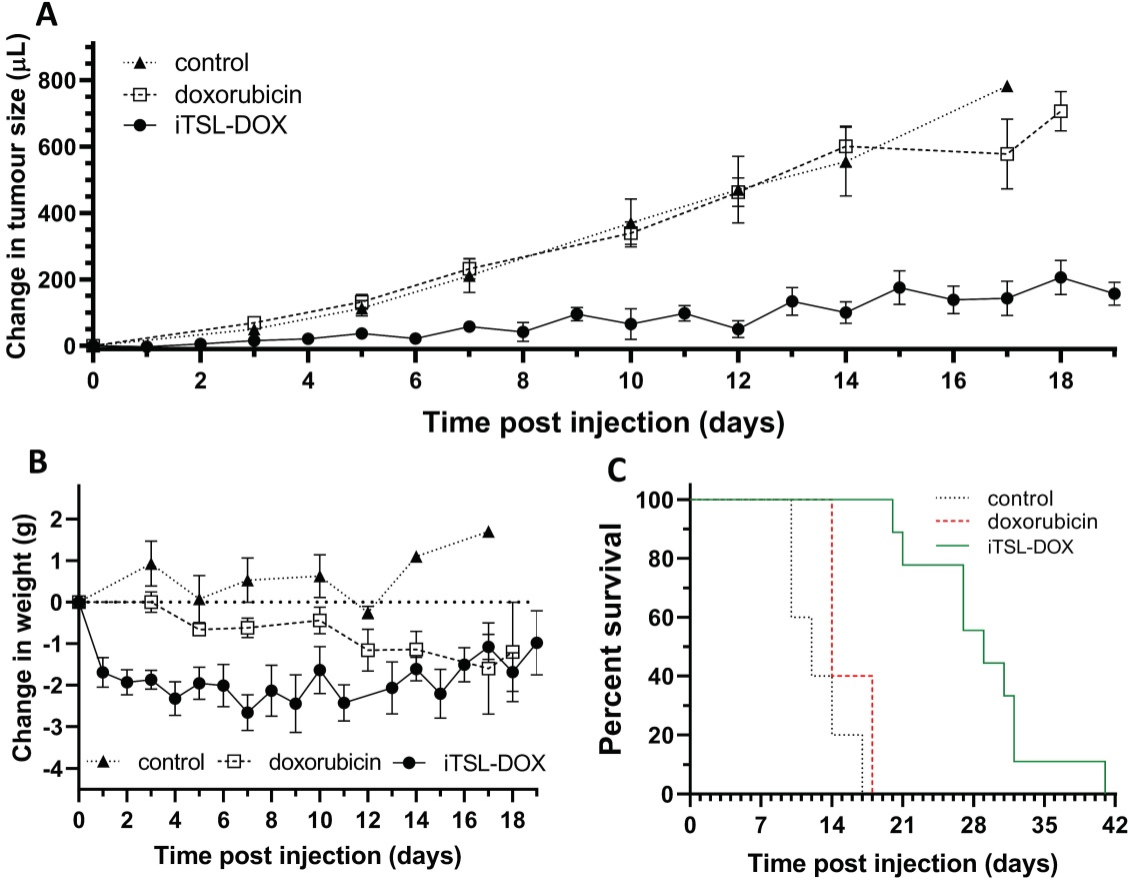
** Single-tumour mouse studies,** showing **(A)** averaged tumour volumes (mean & 1 SEM) for the control (nil drug; n = 5), doxorubicin (n = 5), and iTSL-DOX treated (n = 9) groups; **(B)** body weights and; **(C)** Kaplan-Meier plots for the same, showing survival. Dosage was 4 mg/kg equivalent of doxorubicin and focused ultrasound was applied before/after injection.

Figure 9A presents the tumour growth effects of iTSL-DOX in combination with the brief FUS treatment protocol, compared to controls of doxorubicin or nil-drug (both without FUS). We can observe that FUS in combination with iTSL-DOX induces a powerful retardation in TNBC tumour growth. By contrast, little effect is seen in the group treated with dose-matched doxorubicin alone. Figure 9B shows changes of body weight, with the mice initially at ~22 g. Both drug-treated groups showed weight loss, with the doxorubicin-only mice showing gradual but continual weight reduction over 2 weeks post-injection. Mice treated with iTSL-DOX+FUS initially lost ~10 % (Home Office license limit being 20% of initial body weight) but then stabilised and appeared to partially recover. Overall, mice that were treated with iTSL-DOX+FUS responded better to treatment and survived longer as seen by the Kaplan-Meir curve at Figure 9C.

This breast cancer tumour model was previously used with TSL-DOX and Lokerse *et al.* reports similar levels of tumour growth inhibition at higher dose of 5 mg/kg doxorubicin 52. However, in Lokerse's study hyperthermia (non-focused, the authors did not apply FUS) was applied for a substantially longer time of 60 min. We achieved similar effects with 1/10^th^ of the hyperthermia application time and in a focused manner. Clinical MR-imaging could be of significant assistance here, providing information for location and required thermal dose. A related concern is that extended FUS treatments are harder to translate to the clinic, requiring a patient to spend a significant time immobilised in the scanner 45. Apart from the discomfort, this has important implications on heating precision. Precise hyperthermia for deep seated tumours requires minimised target motion, suggesting that brief repeated rounds may be more practical than 1 h long treatments. Our current data indicate that if frequently repeated, even briefer FUS rounds would benefit precise and efficient treatment.

The effect of FUS-enhanced iTSL-DOX treatments was extended to the double-tumour model (Figure S8). As with the single tumour experiments, iTSL-DOX dosing causes a pronounced reduction in the tumour growth. This occurs even on the nil-FUS tumours but the most significant growth inhibition occurred when iTSL-DOX was combined with FUS. In this study mice also did not show critical weight loss even if they were bearing two tumours. The double tumour mouse model helps in assessing the effect of FUS in combination with the drug, as both tumours receive the dosing the same way and the effect of FUS can be seen more clearly.

**Figure 10:**
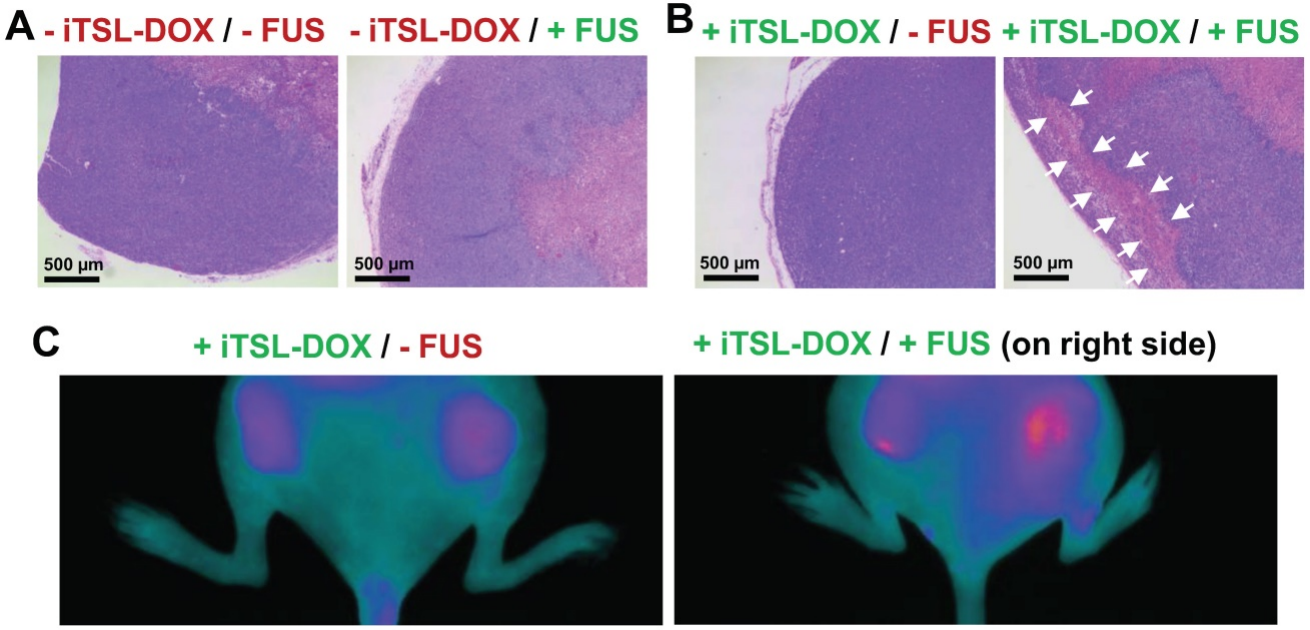
** Assessing necrosis and apoptosis 48 h after treatment. (A)** Histology (H & E sections), tumours were excised 48 h after the treatment. Slices (4 µm) of MDA-MB-231 tumours untreated and **(B)** treated with iTSL-DOX and focused ultrasound (FUS) were stained with haematoxylin and eosin. The necrotic areas induced by the treatment are indicated by the arrows. Large necrotic areas could only be found when the combination of iTSL and hyperthermia was applied; **(C)** Apoptosis monitoring in living mice two days post treatment using *Annexin vivo* 750, with images recorded 3-4 h after the administration. Significant increases in apoptosis were apparent on the right side where FUS had been applied.

Finally we investigated the effects on tumour apoptosis using double-tumour mice. The first group were injected with *Annexin vivo* 750 at 48 h after treatment with iTSL-DOX ± FUS (omitting CF750.DSA to avoid fluorescence interference). Uptake of the apoptotic marker was assessed by NIRF imaging. The second received no marker but were sacrificed at the same time point with tumours excised, mounted, and H & E stained according to standard protocols 53. Figure 10A shows one typical section of completely untreated tumour and one of FUS-only treated tumour. The stained nuclei indicate that the tumours were still viable but a necrotic tumour core is observed. Figure 10B demonstrates sections of tumours treated with iTSL-DOX with or without FUS. In all sections there were areas where nuclei were absent or unstructured (marked with arrows). These were observed at the periphery of the tumour with patches of dead cells forming evident areas of necrosis. Similarly, *Annexin vivo* 750 stained the FUS-treated tumour to a larger effect compared to the FUS-untreated tumour (Figure 10C), indicating that combination of iTSL-DOX and FUS enhanced tumour cell apoptosis. From the above *in vivo* studies it appears that iTSL-DOX released the drug rapidly and this high local dose was efficient in killing tumour cells.

Theranostic liposomes have repeatedly appeared in the literature for MR, PET, and SPECT imaging, as platforms for pH- and temperature-response triggered drug delivery, and for photodynamic or photothermal therapies 54. These functional liposomes are likely the best developed nanomedicine but as functionalities increase there is a need for more sophisticated design. Anti-cancer nanomedicine has shown significant advances in the clinic over the last years with liposomal drugs reaching Phase-III trials 55,56. As yet no MR-labelled or theranostic liposome has reached clinic but a MR-labelled polysiloxane gadolinium macrocycle has recently progressed to Phase-Ib for imaging and radiosensitisation - indicating the potential of these theranostic nanomedicines 57.

Thermosensitive liposomal doxorubicin in the form of ThermoDox® is the most advanced responsive nanomedicine in clinical development. ThermoDox® was first-in-class but has not been as successful in clinic as was expected. Recent clinical trials have combined it with RFA (radiofrequency ablation) for liver tumours but in July 2020, Celsion announced that an independent Data Monitoring Committee recommended halting the Phase-III OPTIMA (NCT02112656) trial although the patients will be followed for overall survival. There may be several reasons for this apparent miss of OPTIMA's primary end point. One potential explanation is that since RFA ablates tumours, it may not be the best hyperthermia induction method to demonstrate clinical efficacy. FUS can offer controlled hyperthermia and when combined with MRI, the targeted tissue temperature is tracked during the treatment. ThermoDox® is planned to be combined in a clinical trial with FUS. This combination of ThermoDox® with a non-invasive hyperthermia technique may restore the clinical efficiency of TSL that is currently under question.

In this study we introduced MR-labelling onto ultrasound responsive liposomes to better control the timing of the thermally-triggered drug release. For this we synthesised a novel MR-lipid and took special attention to use a macrocyclic Gd^3+^ chelator that is considered to be substantially safe and it is widely used in clinical imaging 58. We assessed the gadolinium retention under challenging conditions, and we evidence for the first time that these iTSL do not suffer significant Gd^3+^ leakage under *in vivo* relevant conditions and time frames. MRI studies with tumour-bearing mice demonstrated increased tumour T_1_ contrast for several hours post-injection of iTSL, suggesting that MR-monitoring of iTSL-DOX uptake into tumours will be feasible in the clinic. We also confirmed iTSL-DOX storage stability; a parameter critical for experimental product validation and for clinical translation.

Doxorubicin was selected as the therapeutic cargo. It is a hydrophilic drug, with a good ability to permeate membranes and distribute between tissue compartments 59. It is rapidly cleared from tissues and blood when it is not encapsulated in liposomes. Although several studies have demonstrated the effect of hyperthermia-enhanced doxorubicin uptake by tumours, there is little or no information on clearance from FUS-treated versus non-treated tumours. In our experiments, NIRF imaging suggests that released doxorubicin does not remain in tumour tissue for long, so repetition of FUS rounds might benefit local dosing. Equivalent results were observed in our reported study for iTSL-topotecan 21. Overall it seems iTSL+FUS has the potential to deliver drugs efficiently and specifically, and it is likely that a more potent anticancer agent would better suit this triggered release technology.

## Conclusions

In summary, we have prepared novel MR-imageable liposomes with triggerable release properties, for precise FUS-mediated spatiotemporal tumour drug delivery with direct clinical relevance. The suggested iTSL-DOX is able to enhance MR T_1_ contrast of tumours in mice and provide a good signal for liposome tracking, which in turn allows for timely FUS application. The study shows that real-time imaging and brief FUS insonation combine to assist anticancer treatment to be precisely and correctly released such that a strong anti-tumour effect is achieved. This strategy has the potential advantages of (i) lowering the systemically administered chemotherapeutic dose, as the tumour fraction is dramatically increased; and (ii) providing tumour lesion contrast enhancement while being treated in real time. iTSL is a novel and versatile theranostic platform that could substantially improve chemotherapies' therapeutic index.

## Supplementary Material

Supplementary figures and tables.

## Abbreviations

CA: magnetic resonance imaging contrast agent
CF750: a near-infrared fluorescence dye
CF750.DSA: *N*`-CF750-*N*,*N*-distearylamidomethylamine
DOTA: 1,4,7,10-tetraazacyclododecane-1,4,7,10-tetraacetic acid
DPPC: 1,2-dipalmitoyl-*sn*-glycero-3-phosphocholine
DSC: differential scanning calorimetry
DSL: dynamic light scattering
DSPC: 1,2-distearoyl-*sn*-glycero-3-phosphocholine
DSPE-PEG^2000^: (*ω*-methoxy-polyethyleneglycol^2000^)-*N*-carboxy-1,2-distearoyl-*sn*-glycero-3-phosphoethanolamine
DTPA: 2-[Bis[2-[bis(carboxymethyl)amino]ethyl]amino]acetic acid
ESI: electrospray ionisation
FBS: fetal bovine serum
FDA: food and drug administration
FOV: field of view
FUS: focused ultrasound
Gd.DOTA.DSA: gadolinium (III) 2-(4,7-bis-carboxymethyl-10-[(*N*, *N*-distearylamidomethyl-N'-amidomethyl]-1,4,7,10-tetraazacyclododec-1-*yl*) acetic acid
HEPES: 2-[4-(2-hydroxyethyl)piperazin-1-*yl*]ethanesulfonic acid
HIFU: high intensity focused ultrasound
iTSL: imageable thermosensitive liposome
iTSL-DOX/NO-DOX: imageable thermosensitive liposome with/without doxorubicin
LC-MS/MS: liquid chromatography with tandem mass spectroscopy
LED: light emitting diode
MR: magnetic resonance
MSPC: 1-stearoyl-*sn*-glycero-3-phosphocholine
MWCO: molecular weight cut-off
NIRF: near-infrared fluorescence
MRgFUS: magnetic resonance imaging guided focused ultrasound
PDI: polydispersity index
RO: reverse osmosis
ROI: region of interest
RT: room temperature
SHO: severe combined immunodeficiency hairless outbred
TNBC: triple-negative breast cancer
TSL: thermosensitive liposomes
TXRF: total-reflection X-ray fluorescence
