# Supplementary material for: MR-labelled liposomes and focused ultrasound for spatiotemporally controlled drug release in triple negative breast cancers in mice
Source: Nanotheranostics. 2021 Jan 1;5(2):125–42. doi: 10.7150/ntno.52168 (PMC7806456; doi:10.7150/ntno.52168)
Supplement: Supplementary file 1 — Supplementary figures and tables. [file ntnov05p0125s1.pdf]

Supplementary Materials

MR-labelled liposomes and focused ultrasound for spatiotemporally controlled drug release in triple negative breast cancers

Maral Amrahli, Miguel Centelles, Paul Cressey, Martynas Prusevicius, Wladyslaw Gedroyc, Xiao Yun Xu, Po-Wah So, Michael Wright, and Maya Thanou

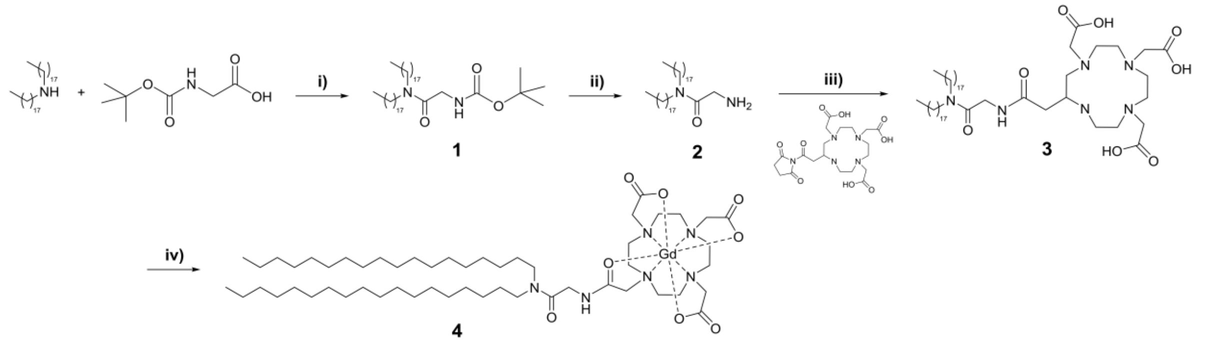

Scheme S1. Synthesis of Gd.DOTA.DSA. Reagents and conditions: (i) dioctadecylamine, HBTU, DMAP, OH.Gly.NH.Boc, dry $CHCl_3$, RT; **1** = 90 %; (ii) TFA: DCM (3:7), RT; **2** = 95 %; (iii) NHS-DOTA, TEA, dry CH_2Cl_2, 35 ^{o}C; **3** = 76 %; (iv) $GdCl_3{\cdot}6H_2O$, H_2O, slow reflux; **4** = 82 %. **2. DSA.** ^{1}H (400 MHz; $CDCl_3$; 296 K) δ 3.84 (s, 2H, $OC\underline{CH}_2NH_2$), 3.29 (t, J = 8.0 Hz, 2H, $OCNCH_2$), 3.11 (t, J = 7.8 Hz, 2H, $OCNCH_2$), 1.50 (m, 4H, $OCNCH_2C\underline{H}_2$), 1.25 (s, 60H, alky chain CH_2), 0.88 (t, J = 6.3 Hz, 6H, CH_3); ^{13}C (100 MHz; CD_2Cl_2; 296 K) δ 166.6 (OCN) 48.8 & 48.1 ($OCN\underline{C}H_2$), 41.6 ($OC\underline{C}H_2NH_2$), 31.1 ($CH_3CH_2\underline{C}H_2$), 30.9-30.8 (alkyl chain CH_2), 29.9 ($OCNCH_2CH_2CH_2\underline{C}H_2$), 28.8-28.3 ($OCNCH_2\underline{C}H_2\underline{C}H_2$), 24.2 ($CH_3\underline{C}H_2$), 15.4 ($CH_3$). TLC (15% MeOH in CH_2Cl_2 with 0.5 % NH_3) gave R_f 0.55 with the DSA spot showing red after sequential vanillin and ninhydrin stains. ESI-MS calcd. for $C_{38}H_{78}N_2O$ $[M+H]^+$: 579.8 a.m.u. Found $[M+H]^+$ 579.7 a.m.u. **3. DOTA.DSA.** ^{1}H (400 MHz; $CDCl_3/CD_3OD$) δ 3.45 (br, 2H, $NC\underline{H}_2CONH$), 3.10 (br, 6H, $NC\underline{H}_2COOH$), 3.00 (br, 2H, $OCNC\underline{H}_2$), 2.80 (br, 16H, $NC\underline{H}_2C\underline{H}_2N$), 2.28 (br, 2H, $OCNC\underline{H}_2$), 2.16 (br, 2H, $OCC\underline{H}_2NH$), 1.44 (m, 4H, $OCNCH_2C\underline{H}_2$), 1.18 (s, 60H, alky chain CH_2), 0.80 (t, J = 6.6 Hz, 6H, CH_3). ^{13}C (100 MHz; CD_2Cl_2/CD_3OD; 296 K) δ 47.0 ($OCN\underline{C}H_2$), 41.5-38.5 ($N\underline{C}H_2\underline{C}H_2N$ & $N\underline{C}H_2COOH$), 32.3 ($CH_3CH_2\underline{C}H_2$), 31.5-28.5 (alkyl chain CH_2), 22.9 ($CH_3\underline{C}H_2$), 14.1 (CH_3); others could not be distinguished. ESI-MS calcd. for $C_{54}H_{104}N_6O_8$ $[M+H]^+$: 965.8 a.m.u. Found $[M+H]^+$ 965.7 a.m.u with major fragments seen at 579.6, 522.3, 444.1 and 387.1 a.m.u. corresponding to **2**, $(C_{18})_2NH$, DOTA-glycine and DOTA respectively. **4. Gd.DOTA.DSA.** The presence of Gd disrupted the ^{1}H and ^{13}C NMR spectra. ESI-MS calcd. for $C_{54}H_{101}GdN_6O_8$ $[M+H]^+$: 1120.7 a.m.u. Found $[M+H]^+$ 1120.6 a.m.u. with major fragments seen at 1076.5, 1032.5, 1005.7, and 988.8 a.m.u. (all + p ESI) corresponding to loss of COO, 2x COO, 2x CH_2COO, and 3x COO respectively.

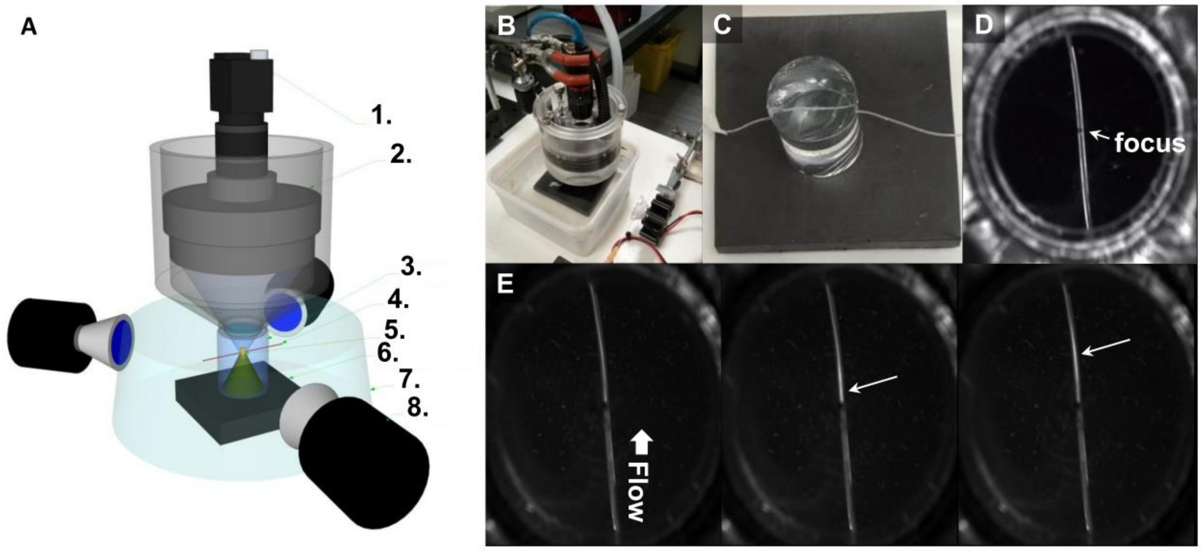

Figure S1. Assessing doxorubicin release under FUS, measured by intrinsic doxorubicin fluorescence; **(A)** schematic showing a polyacryamide gel embedded flow-tube, light source and camera, around a FUS system; 1. camera, lens, and filter; 2. transducer; 3. gel block; 4. flow tube; 5. focus; 6. acoustic foam; 7. water bath; 8. LED lights; **(B)** photographs of the setup around the transducer; **(C)** close up of the gel block and flow tube; **(D)** view showing the flow-tube and an indication of the FUS focus with the fine-wire thermocouple visible in reflection. Pulsed FUS insonation of a flowing iTSL stream then causes synchronised fluorescence intensity increases, indicating boluses of released doxorubicin; **(E)** Three representative frames showing (left to right) FUS-off, start of FUS and fluorescence increase, and FUS-off again and wash out of the release doxorubicin bolus.

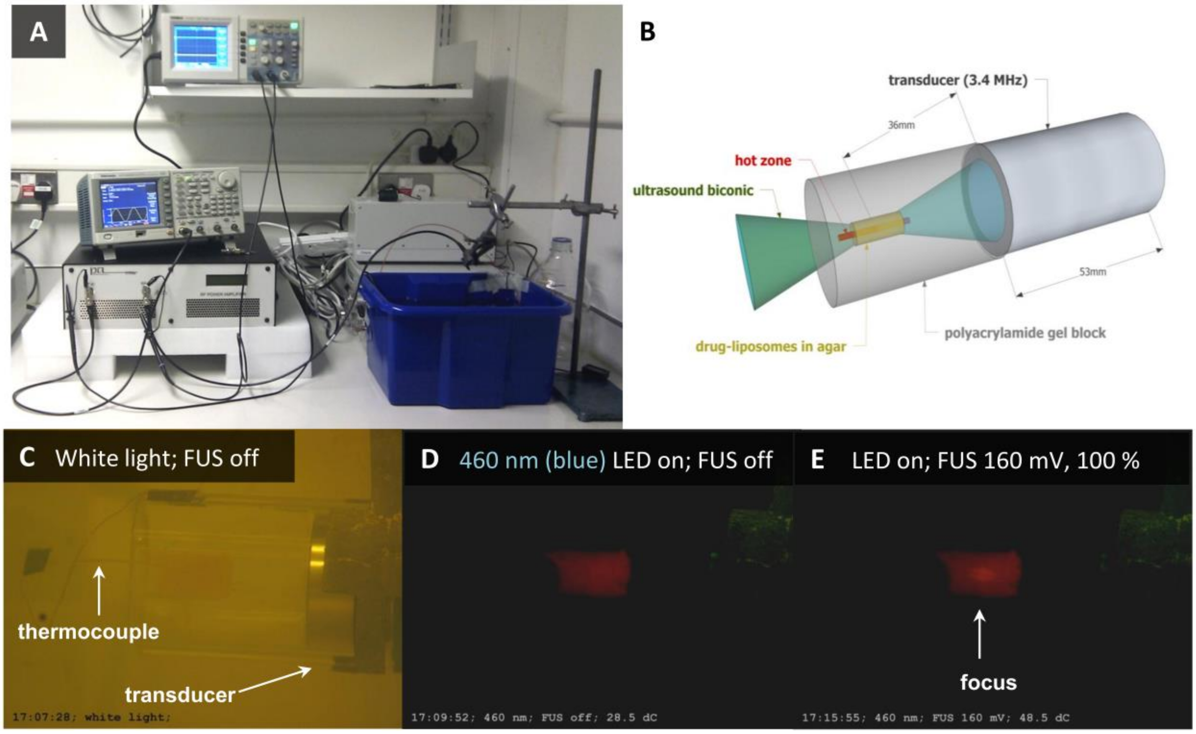

Figure S2. Supporting data for the assessment of FUS-induced doxorubicin release: (A, B) this prototype used a 3.4 MHz FUS transducer (Precision Acoustics, UK) focused on iTSL-DOX / agar phantoms embedded in a larger polyacrylamide gel block. Low gel-point agar allowed the iTSL-DOX to be immobilised without heating above the T_m, while the polyacrylamide allow for placement and retention without interfering with FUS transmission. Focus temperature was measured with a fine-wire thermocouple and doxorubicin release by intrinsic fluorescence using a monochromatic LED (460 nm) source, combined with a domestic camera with glass photographic (‘G’) filters and video collection settings. This approach was sufficient for a proof-of-principle but had limitations of poor spectral specificity and low frame rates (6-8 fps). Example frames showing **(C)** embedded iTSL-DOX/agar as a pale pink cylinder under white light. The encasing polyacrylamide is optically clear, while the supporting acrylic cylinder can be seen, along with the transducer face on the right; **(D)** Under blue light but no FUS the encapsulated doxorubicin shows deep-red fluorescence; **(E)** This significantly increases in brightness once the FUS is turned on (160 mV input to amplifier; 100 % duty cycle). This change is irreversible and demonstrates the fluorescence de-quenching seen from iTSL-released doxorubicin.

See also: http://youtu.be/N6N6GgY49CA

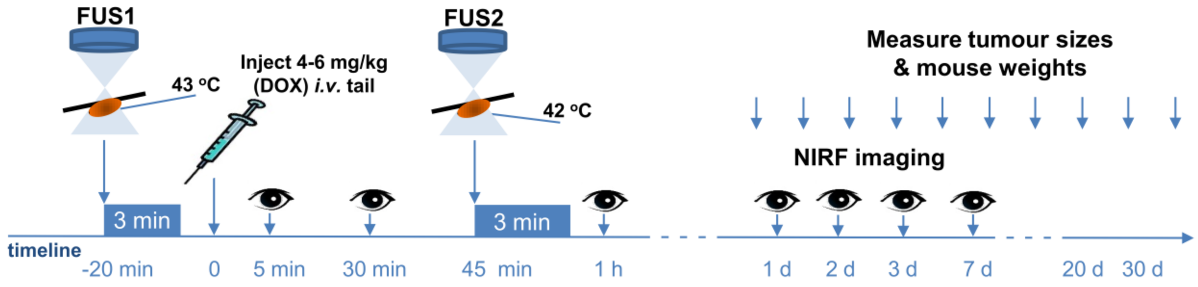

Figure S3. **Preclinical FUS studies; Study outline** - once tumours were ~ 5 mm each animal received: (i) a leading FUS treatment (42-43 ^{o}C; 3 min); (ii) injection of iTSL-DOX at t = 0; (iii) a second FUS treatment (42 ^{o}C for 3 min) applied once imaging observed iTSL-DOX had accumulated in the tumour; (iv) monitoring by whole body NIRF, tumour sizing, and weight measurement until the end of the study.

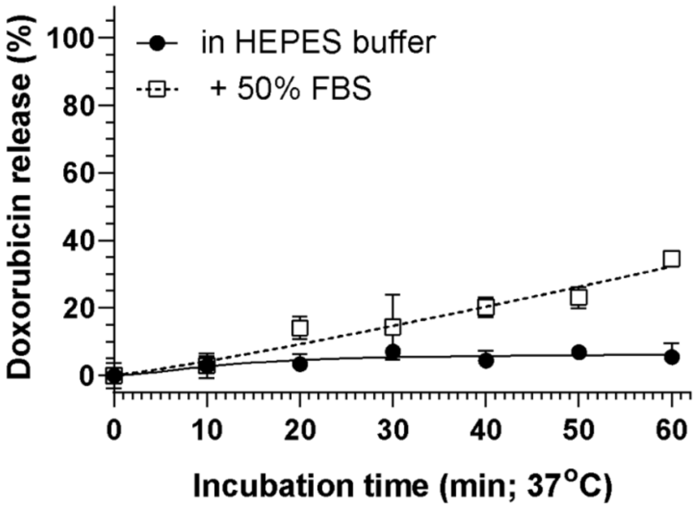

Figure S4. Effect of serum on doxorubicin release from iTSL-DOX samples incubated at 37 ^{o}C for up to 60 min in buffer (50 mM HEPES with 5 w% glucose; pH 7.4), compared with buffer containing 50 v% foetal bovine serum (FBS) as a blood analogue. Release was monitored by the increase of intrinsic doxorubicin fluorescence (Ex_{480} / Em_{590} nm) as it leaves the self-quenched encapsulated state. N = 3, values are mean ± SD.

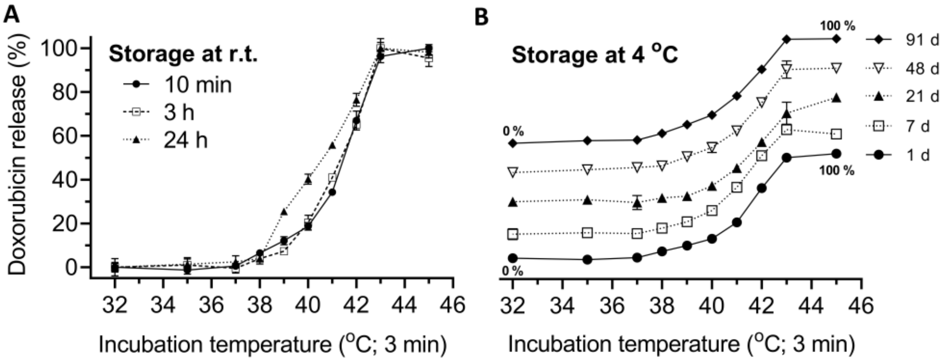

C

day	Size (nm)	PDI
1	180.4	0.225
4	183.1	0.206
7	173.9	0.225
19	177.3	0.219
21	177.4	0.198
28	180.6	0.211
34	182.5	0.190
41	176.9	0.219
48	177.5	0.236
62	180.0	0.213

Figure S5. Storage stability of iTSL-DOX; Doxorubicin release was studied by incubating samples for 3 min at 32-46 ^{o}C. The graphs show %release for stocks either left at **(A)** room temperature for 10 min, 3 h, or 24 h (n = 3; mean ± SD) or in; **(B)** cold storage at ~ 5 ^{o}C (stacked curves; n = 3; mean ± SD). Little or no change is seen in the thermal release profiles as the liposomes ages; **(C)** representative average particle diameter and PDI data also shows no significant changes on storage for 2 months.

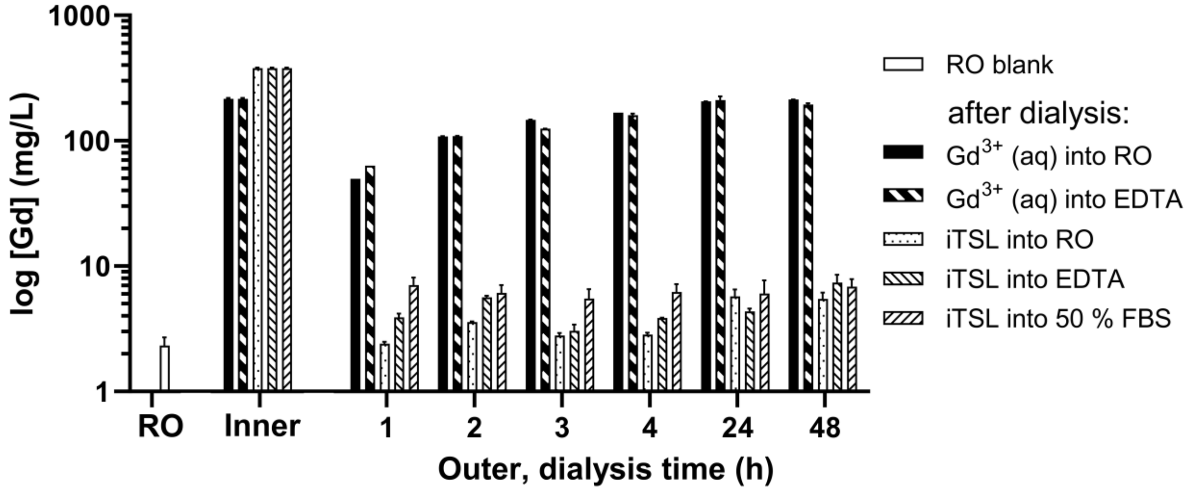

Figure S6. Gadolinium leakage analysis using dialysis membranes and total reflection X-ray fluorescence (TXRF). The potential for loss of the metal from Gd.DOTA.DSA was established by assaying the amount of Gd^{3+} able to escape through a dialysis membrane from an inner chamber containing either 0.2 mg/mL gadolinium standard or iTSL (equivalent to 0.38 mg/mL Gd) and into a cuvette containing reverse osmosis (RO) grade water at RT or 50 % (v/v) fetal bovine serum at 4 °C (to avoid serum degradation). The cuvettes were placed on a magnetic stirrer and 10 µL samples were taken at 1-48 h time points. These were analysed to determine the concentration of gadolinium (n = 3; mean ± SD). A scaled baseline is also given for n = 11 samples of RO water.

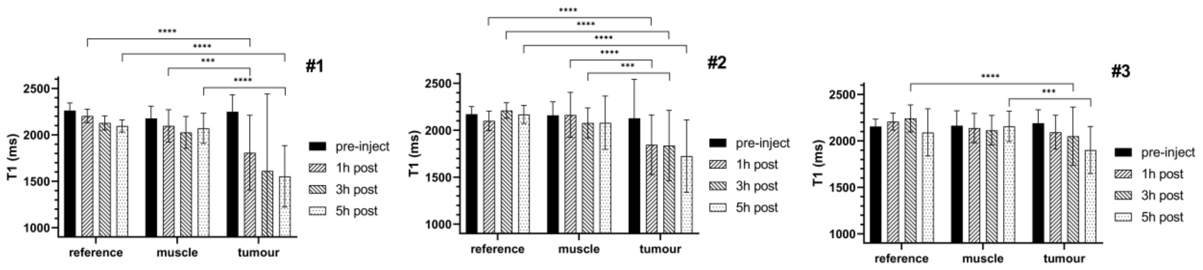

Figure S7. Collated pixel intensities from matched ROIs in all T_1 map slices underwent frequency distribution analysis in Prism (Graphpad Software, San Diego CA, USA) with bin-width 50 over 800-3000 units. The resulting histograms were then non-linear regression fit to Gaussian curves and the resulting best-fit value means and S.D.s (equivalent to the distribution breadth) cross-compared for each animal (n = 3), time-point, and ROI. Significance markers refer to ANOVA 1-way analyses on the collated raw data using default settings: *** P < 0.0002, **** P < 0.0001. Little or no difference is seen from neither the Gadovist reference nor the muscle tissue controls. Significant mean reduction is seen in the majority of tumours immediately post-injection. There is often an increase in the distribution SD, signifying significant heterogeneity. This likely relates to the increased tumour vascularity and/or the presence of a low-infusion core.

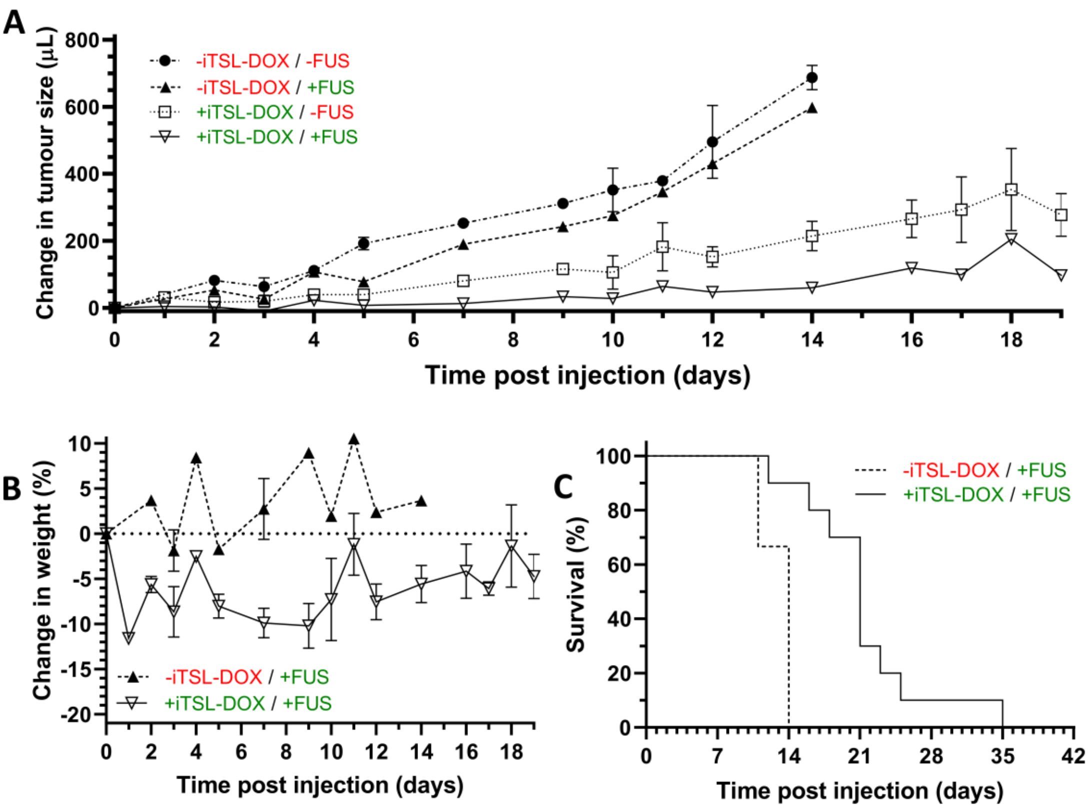

Figure S8. Double-tumour mouse studies with only the right-side tumour treated by FUS. The groups were: nil-drug ± FUS (n = 3) and iTSL-DOX ± FUS (n = 10); **(A)** average tumour sizes (mean ± SEM). Mice were injected (*i. v.* tail) to 6 mg/kg equivalent doxorubicin on day 0 and FUS treatment was applied pre/post injection; **(B)** average body weights and; **(C)** Kaplan-Meier plots showing survival. Weights are given as mean ± SEM.

For these double-tumour studies, mouse survival is limited by the growth of the non-FUS tumour, which receives only a reduced dosage of iTSL-DOX. The approach allows for more direct comparison of the effects of FUS across the two tumours of the same animal but reduces overall survival improvements compared to the single-tumour studies.

Formulation	T_{on}	T_m	T_{cl}
100 mol% DPPC	39.3	41.7	42.8
30 mol% Gd.DOTA.DSA, 70 mol% DPPC	39.4	41.6	42.4
Reference LTSL	40.2	42.1	43.7
iTSL	41.2	43.3	45.8

Table S1. Measurements carried out on a TA Instruments Nano DSC in HEPES/glucose buffer, against a reference of the same. Values are indicative examples with estimated error ± 0.2 ^{o}C. $T_{on/cl}$ are calculated as the first and last temperatures at which the thermal power is 5 % of T_m.
